# Epigenome signatures landscaped by histone H3K9me3 are associated with the synaptic dysfunction in Alzheimer's disease

**DOI:** 10.1111/acel.13153

**Published:** 2020-05-17

**Authors:** Min Young Lee, Junghee Lee, Seung Jae Hyeon, Hyesun Cho, Yu Jin Hwang, Jong‐Yeon Shin, Ann C. McKee, Neil W. Kowall, Jong‐Il Kim, Thor D. Stein, Daehee Hwang, Hoon Ryu

**Affiliations:** ^1^ Institute for Systems Biology Seattle WA USA; ^2^ Veteran's Affairs Boston Healthcare System Boston MA USA; ^3^ Department of Neurology Boston University Alzheimer’s Disease Center Boston University School of Medicine Boston MA USA; ^4^ Center for Neuromedicine Brain Science Institute Korea Institute of Science and Technology Seoul South Korea; ^5^ Genome Medicine Institute and Department of Biochemistry Seoul National University College of Medicine Seoul South Korea; ^6^ Center for the Study of Traumatic Encephalopathy Boston University School of Medicine Boston MA USA; ^7^ Department of Biological Sciences Seoul National University Seoul South Korea

**Keywords:** Alzheimer's disease, epigenetic modifications, genome‐wide sequencing, histone H3K9me3, synaptic transmission

## Abstract

The pathogenesis of Alzheimer's disease (AD) and the commonest cause of dementia in the elderly remain incompletely understood. Recently, epigenetic modifications have been shown to play a potential role in neurodegeneration, but the specific involvement of epigenetic signatures landscaped by heterochromatin has not been studied in AD. Herein, we discovered that H3K9me3‐mediated heterochromatin condensation is elevated in the cortex of sporadic AD postmortem brains. In order to identify which epigenomes are modulated by heterochromatin, we performed H3K9me3‐chromatin immunoprecipitation (ChIP)‐sequencing and mRNA‐sequencing on postmortem brains from normal subjects and AD patients. The integrated analyses of genome‐wide ChIP‐ and mRNA‐sequencing data identified epigenomes that were highly occupied by H3K9me3 and inversely correlated with their mRNA expression levels in AD. Biological network analysis further revealed H3K9me3‐landscaped epigenomes to be mainly involved in synaptic transmission, neuronal differentiation, and cell motility. Together, our data show that the abnormal heterochromatin remodeling by H3K9me3 leads to down‐regulation of synaptic function‐related genes, suggesting that the epigenetic alteration by H3K9me3 is associated with the synaptic pathology of sporadic AD.

## Graphical abstract

Histone H3K9me3 is densely stained in nuclear foci where heterochromatins are spatially localized in neurons of AD temporal cortex. H3k9me3‐heterochromatin‐landscaped epigenomes are closely associated with the synaptic dysfunction in AD. Together, the epigenetically disrupted biological pathways could serve as novel biomarkers in AD.

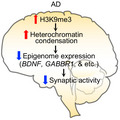

## 1 INTRODUCTION

Alzheimer's disease (AD), the most common progressive neurodegenerative disease, is the leading cause of dementia in the elderly (Querfurth & LaFerla, 2010). The genetic and environmental factors responsible for AD may impact the expression of thousands of genes involved in molecular and cellular functions of the brain (Mastroeni et al., 2011). Except for the Aβ‐related mutations, most genetic factors have been shown to have low penetrance, and many individuals with salient risk factors are not affected (Lue, Brachova, Civin, & Rogers, 1996). Although there are genetic effects on the pathogenesis of familial AD, most AD cases are not familial and do not have a simple genetic cause suggesting that environmental factors are important to AD pathogenesis. Interestingly, monozygotic twins have dichotomous phenotypes of AD. In monozygotic twin cohort studies, AD has been associated with environmental factors, such as advanced maternal age, head trauma, history of depression, and manual work (Raiha, Kaprio, Koskenvuo, Rajala, & Sourander, 1998).

Recently, histone modifications, DNA methylation, ribosomal DNAs (rDNAs), and microRNAs (miRNAs) have been suggested as epigenetic factors mediating the influence of environmental factors on AD‐related gene expression. For example, treatment with valproic acid, a histone deacetylase 1 (HDAC1) inhibitor, was shown to decrease Aβ production in the brains of *PDAPP* (APP (V717F)) transgenic mice (Su et al., 2004). Further, elevated plasma homocysteine, which inhibits DNA methylation, has been reported to be a significant risk factor for AD (Ravaglia et al., 2005; Seshadri et al., 2002). The expression of genes involved in Aβ production, specifically β‐secretase 1 (*BACE1*) and presenilin 1 (*PSEN1*), is increased after folate deprivation‐induced DNA hypomethylation and restored to normal after supplementation with S‐adenosylmethionine (SAM) (Fuso et al., 2008). Trimethylated histone H3K9 (H3K9me3) is one of the key histone modifications associated with decreased transcriptional activity and heterochromatin condensation (Wu, Terry, Singh, & Gilbert, 2005). The methylation of histone H3K9 serves to silence gene expression. We have previously reported that the increased H3K9me3 levels are correlated with transcriptional dysfunction and neurodegeneration in animal models of Huntington's disease (HD) and human HD patients (Ryu et al., 2006; Lee et al., 2017). Levels of trimethylated H3K9 (H3K9me3), in particular, have been correlated with gene silencing and condensation of constitutive heterochromatin. Importantly, this single modification in H3K9 is directly associated with altered chromatin plasticity under neurodegenerative conditions (Lee et al., 2013; Ryu, Barrup, Kowall, & McKee, 2008). In a preliminary study, we found that H3K9me3 levels were markedly increased in the anterior temporal neocortex and hippocampus in monozygotic twin pairs discordant for AD (Ryu et al., 2008). However, H3K9me3‐dependent gene silencing and the transcriptome specifically targeted by H3K9me3 have not been systematically explored in sporadic cases of AD.

We aimed the current study to examine whether H3K9me3‐dependent heterochromatin remodeling is altered and which H3K9me3‐associated epigenome signatures are associated with the pathogenesis of sporadic AD. We performed an integrated analysis of H3K9me3‐chromatin immunoprecipitation (ChIP)‐sequencing (ChIP‐seq) and mRNA‐sequencing (mRNA‐seq) on brain samples obtained from AD patients and normal subjects. This analysis was performed to determine how heterochromatin remodeling driven by H3K9me3 is altered in AD and to define the epigenome profiles landscaped by H3K9me3 and linked to AD pathogenesis. Network analysis discovered that H3K9me3‐landscaped epigenomes are associated with synaptic dysfunction. The H3K9me3‐enriched epigenomes* *were further verified using qPCR on brain tissue from normal and AD subjects. Our data show that alterations of chromatin remodeling and gene expression landscaped by H3K9me3 contribute to AD pathogenesis.

## 2 RESULTS

### 2.1 H3K9me3 immunoreactivity, a mark of heterochromatin structure, is elevated in AD

**FIGURE 1:**
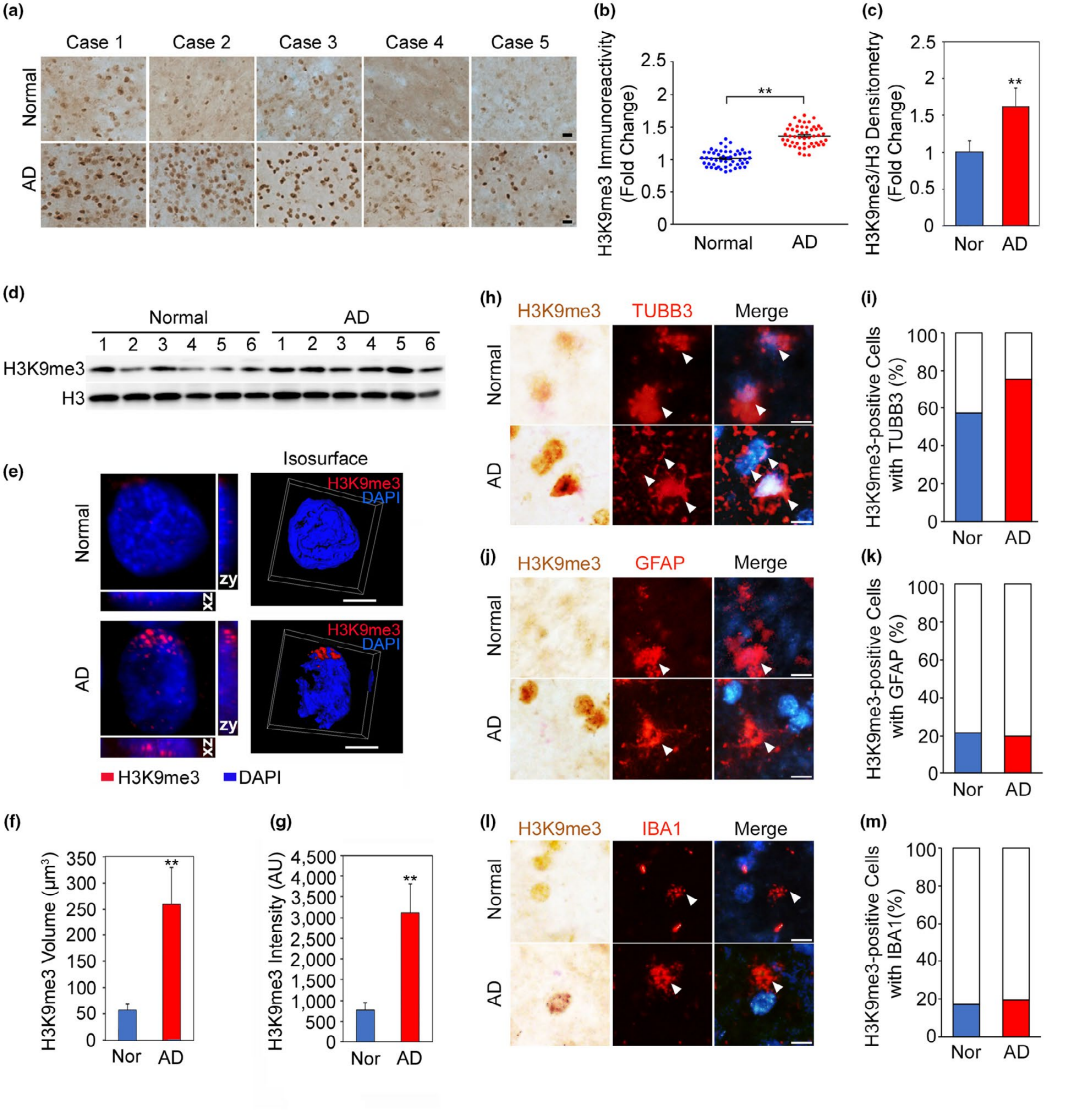
H3K9me3‐dependent heterochromatin is altered in the cortical neurons of AD. (a) H3K9me3 immunoreactivity was increased in the cortical neurons of AD compared to normal subjects. Scale bars (black): 20 µm. (b) The densitometry analysis showed a significant increase of H3K9me3 immunoreactivity in the cortical neurons of AD [cases, *N* = 5; cell counting, *n* = 50 (10 cells/case)] compared to normal subjects [cases, *N* = 5; cell counting, *n* = 50 (10 cells/case)]. H3K9me3 levels were normalized to histone H3. **Significantly different at *p* < .001. (c) The densitometry analysis of Western blot analysis showed a significant increase of H3K9me3 levels in AD (*N* = 9) compared to normal subjects (*N* = 9). H3K9me3 levels were normalized to histone H3. **, Significantly different at *p* < .001. (D) A representative Western blot showing elevated H3K9me3 levels in the temporal cortex of AD patients (*N* = 6) compared to normal subjects (*N* = 6). (e) Three‐dimensional (3D) reconstruction of confocal fluorescent micrographs and isosurface images illustrate that spatially condensed patterns of H3K9me3‐positive heterochromatin structures (red) are increased eccentrically in AD. The nuclei were counter stained with DAPI (blue). Scale bars (white): 8 µm. (f, g) Both the volumetric expansion and the intensity of H3K9me3‐dependent heterochromatin are significantly increased in AD. **Significantly different at *p* < .001. (h) H3K9me3 immunoreactivity was colocalized with the neuron marker (TUBB3) in the cortex of AD brain and normal subjects. (i) The counting analysis showed a significant increase of H3K9me3 immunoreactivity in the TUBB3‐positive cortical neurons of AD. (j) H3K9me3 immunoreactivity was not highly colocalized with the astrocyte marker (GFAP) in the AD and normal subjects. (k) The counting analysis showed no significant changes of H3K9me3 immunoreactivity in the GFAP‐positive astrocytes in the cortex of AD. (l) H3K9me3 immunoreactivity was not highly colocalized with the microglial marker (IBA1) in the AD brain and normal subjects. Scale bars (white): 20 µm. (m) The counting analysis showed no significant changes of H3K9me3 immunoreactivity in IBA1‐positive microglia in the cortex of AD

In order to examine whether elevated H3K9me3 is associated with abnormal heterochromatin structure in AD, we first performed immunohistochemistry. As expected, the immunoreactivity of H3K9me3 was robustly elevated in the temporal cortex of sporadic AD patients (*N* = 5) compared to normal subjects (*N* = 5; Figure 1a). The densitometry analysis showed a significant increase of the H3K9me3 levels in the cortical neurons of AD brains (Figure 1b). Next, we measured H3K9me3 levels by Western blot analysis in the temporal cortex of AD and normal subjects. The densitometry analysis verified that H3K9me3 levels were significantly increased in AD patients (*N* = 9) compared to normal subjects (*N* = 9; Figure 1c,d). Additionally, we performed immunofluorescence staining combined with confocal microscopy analysis. We found that H3K9me3 (red color) is localized within dense nuclear foci of neuronal chromatin (blue color stained with DAPI) in the temporal cortex of AD (Figure 1e). The majority of H3K9me3 immunoreactivity was eccentrically localized, indicating that the H3K9me3‐dependent heterochromatin foci in AD brains are spatially distinctive from controls. In order to further analyze the microstructure of H3K9me3‐dependent heterochromatin structure, we deconvolved confocal images and analyzed spatial patterns for the expansion of H3K9me3‐positive heterochromatin condensation between normal and AD (Figure 1e). Three‐dimensional deconvolution and isosurface image analysis illustrated clearer localization of H3K9me3‐dependent heterochromatin condensation in the cortical neurons of AD (Figure 1e). Interestingly, the H3K9me3‐dependent heterochromatin structures were unevenly distributed and polarized to one side of the nucleus. In addition, quantitative image analysis showed a fivefold increase in the volume of H3K9me3‐dependent heterochromatin and a fourfold increase in the intensity of H3K9me3‐dependent heterochromatin in AD compared to normal (Figure 1f,g).

Furthermore, to determine how H3K9me3 is expressed in neuronal versus non‐neuronal cell types, we performed double chromogenic and immunofluorescence staining with cell‐type‐specific markers such as TUBB3 for neuron, GFAP for astrocyte, and IBA1 for microglia, respectively. As expected, the H3K9me3 immunoreactivity was colocalized with TUBB3‐positive neurons and elevated in the cortical neurons of AD postmortem brains compared to that of normal subjects (Figure 1h,i). Otherwise, the H3K9me3 immunoreactivity was not highly colocalized with GFAP‐positive astrocytes both in the AD brain and normal subjects (Figure 1j). The H3K9me3‐/GFAP‐double positive cells were not significantly altered in the cortex of AD (Figure 1k). Neither the H3K9me3 immunoreactivity was highly colocalized with the IBA1‐positive microglia both in the AD brain and normal subjects nor H3K9me3‐/IBA1‐double‐positive cells were significantly changed in the cortex of AD (Figure 1l,m).

### 2.2 The occupancy of H3K9me3 is differentially marked in AD

**FIGURE 2:**
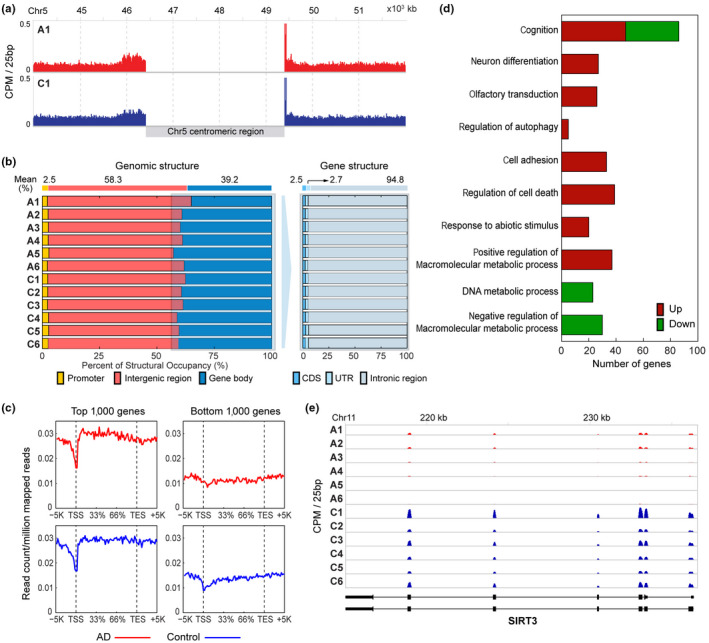
H3K9me3 is differentially marked in AD. (a) Higher occupancy of H3K9me3 in the centromeres of AD (A1) and normal control (C1) brains. See also Figure S1 for the other samples. (b) Genomic structural occupancy of H3K9me3. Right panel shows the proportions of H3K9me3 occupancy in the promoter, intergenic, and gene body regions in six AD (A1‐6) and normal control (C1‐6) brains. Left panel shows the proportions of H3K9me3 gene body occupancy in CDS, UTR, and introns. (c) H3K9me3 occupancy profiles in the structure of genes with high (left) and low (right) levels of H3K9me3 in AD (top) and normal control (bottom) brains. For each gene, the gene structure spanned from 5kb upstream of TSS to 5kb downstream of TES, and the whole region was divided into 100 bins. The mean read counts of the top or bottom 1000 genes with high or low levels of H3K9me3, respectively, were displayed in individual bins. (d) GOBPs (by the DMGs) that were significantly associated with in AD (*p* < .01). The number of genes with increased (red) or decreased (green) levels of H3K9me3 in AD involved in each GOBP is shown. (e) A representative gene with decreased levels of H3K9me3 in AD (A1‐6) compared to normal control subjects (C1‐6). Read counts were displayed along exons and introns shown in the bottom

To discover whether H3K9m3 is differentially marked in AD, we performed H3K9me3‐ChIP‐seq of cortex tissues from six AD and six normal subjects. The clinical information of the postmortem brains was summarized in Table S1. Through the ChIP‐seq, an average of 48.1 million reads were obtained from individual samples, and those reads were aligned to the human genome using bowtie (version 2.0.6). It resulted in 2.8 Giga bps of mapped sequences, equivalent to 0.8‐fold coverage of the annotated human genome (Langmead & Salzberg, 2012; Table S2). At the chromosomal level, H3K9me3 was strongly enriched near the centromeres, typical heterochromatins, in both AD and normal brains (Figure 2a, Figure S1), consistent with previous findings (Barski et al., 2007). Moreover, H3K9me3 was enriched mainly in both noncoding intergenic and intragenic regions (58.3% and 37.1%, respectively; Figure 2b, Table S3, Figure S2). We further compared the distribution of H3K9me3 across the gene structure for the genes with high and low levels of H3K9me3 in euchromatin regions. The top 1,000 genes with the highest levels of H3K9me3 showed that H3K9me3 was highly enriched in gene bodies containing coding DNA sequences (CDS), consistent with previous findings (Cheng et al., 2011) (Figure 2c). In contrast, the bottom 1,000 genes with the lowest levels of H3K9me3 showed low enrichment of H3K9me3 in gene bodies.

Our data showed that H3K9me3 was enriched in gene bodies (CDS) of the genes with high levels of H3K9me3 (Figure S2), in addition to the enrichment of H3K9me3 in the centromere and the noncoding intergenic and intragenic regions. This suggests that gene body H3K9me3 is differentially marked between AD and normal brains. To further examine H3K9me3 in AD, we next identified 1,388 differentially marked genes (DMGs) between AD and normal brains as described in Section 4 (Table S4). To understand cellular processes associated with the AD‐related changes in H3K9me3, we then performed functional enrichment analysis for the DMGs using DAVID software and identified Gene Ontology biological processes (GOBPs) represented by the DMGs (Table S5). The DMGs are mainly involved in cognition, neuron differentiation, cell death, and cell adhesion (Figure 2d), suggesting that H3K9me3 can affect these processes by altering expression of the genes involved in these processes. The decreased H3K9me3 in the gene bodies of SIRT3 in AD was shown in Figure 2e.

### 2.3 Transcriptome is differentially regulated in AD

**FIGURE 3:**
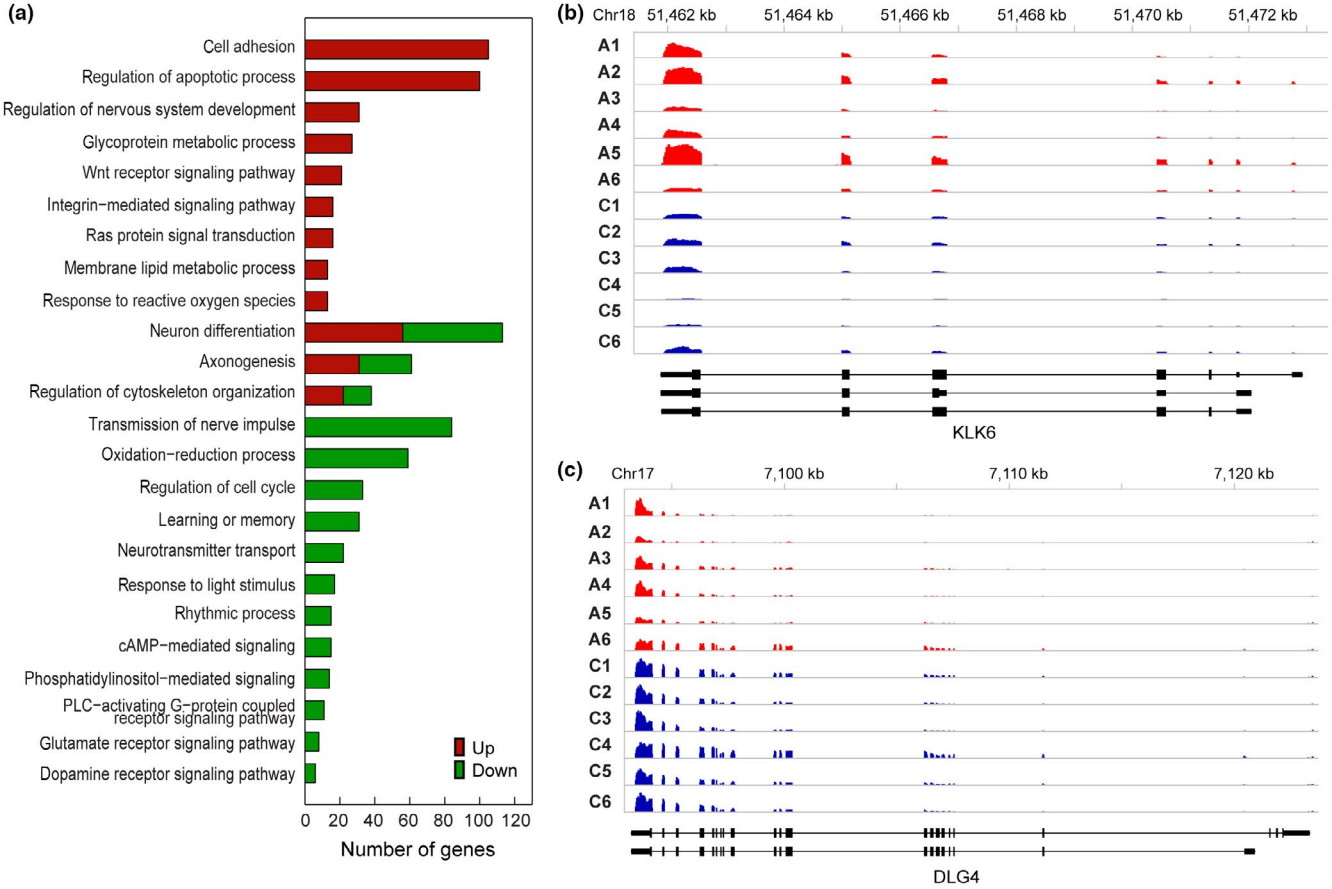
Transcriptome is differentially expressed in AD. (a) GOBPs (by the DEGs) that were significantly associated with AD (*p* < .01). The number of up‐ (red) or down‐regulated (green) genes involved in each GOBP is shown. (b) A representative up‐regulated (*KLK6*) gene in AD patients (A1‐6) compared to normal control subjects (C1‐6). (c) A representative down‐regulated (*DLG4*) gene in AD patients (A1‐6) compared to normal control subjects (C1‐6). For each gene, read counts were displayed along exons and introns shown in the bottom

**FIGURE 4:**
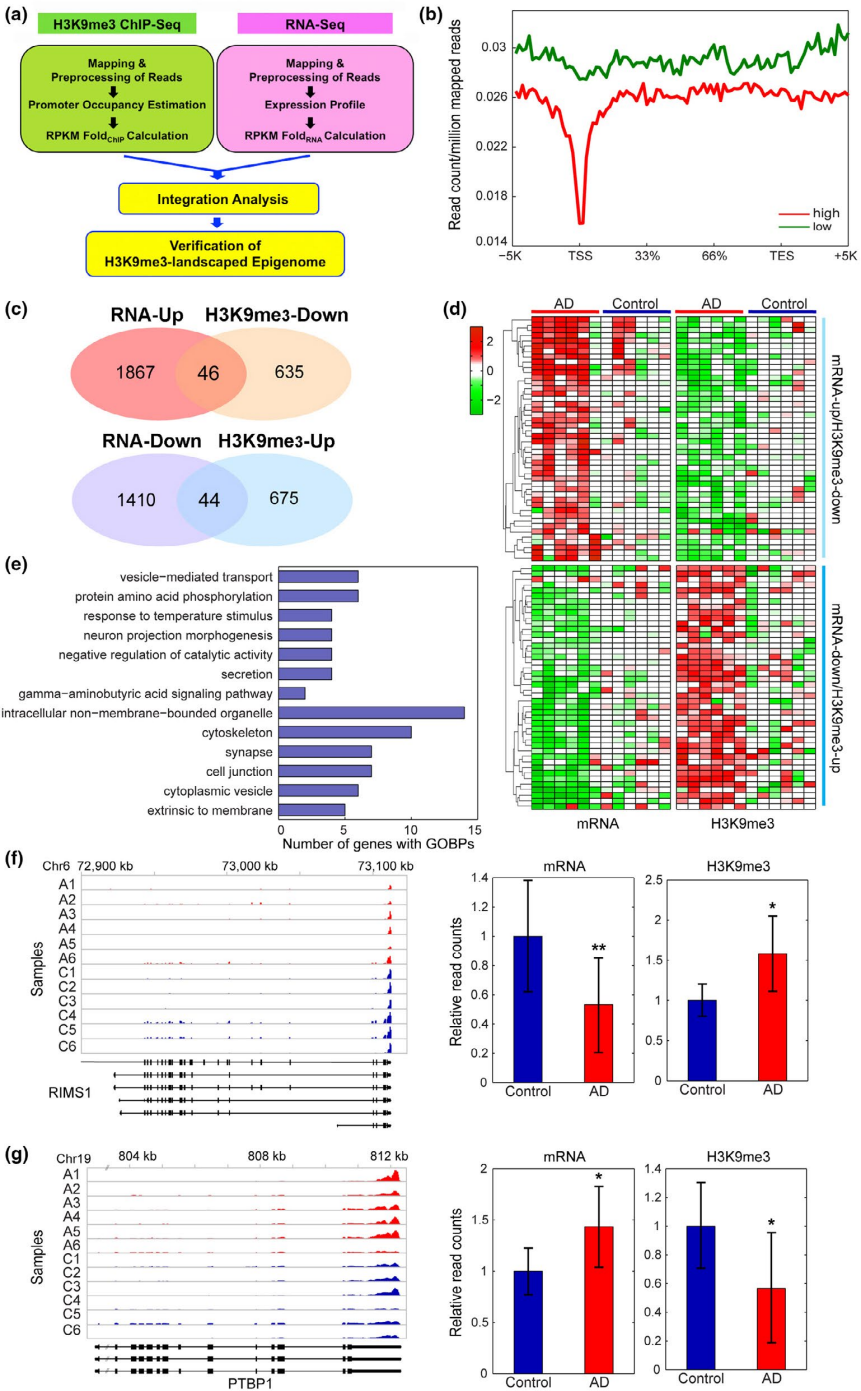
Integrated analysis of genome‐wide ChIP‐ and mRNA‐sequencing identifies alterations of H3K9me3‐enriched transcriptomes in AD. (a) A scheme representing the integration analysis strategy to identify H3K9me3‐landscaped transcriptome signatures in AD patients. (b) mRNA expression stratified profiles of H3K9me3 occupancy in the gene structure. The profiles were calculated as described in Materials and Methods and represented for highly (red) and lowly (green) expressed genes, respectively. (c) The overlapped regions of Venn diagrams showed altered epigenomes whose levels of H3K9me3 and mRNA are inversely correlated (H3K9me3‐Up versus RNA‐Down and vice versa) in AD patients. (d) Heat maps showing the genes with divergent changes of H3K9me3 and mRNA expression levels between AD patients (*N* = 6) and normal subjects (*N* = 6). The dendrogram was generated by hierarchical clustering of H3K9me3 and mRNA expression changes using Ward's linkage and Euclidean distance as a dissimilarity measure. (e) GOBPs analysis representing the epigenomes whose levels of H3K9me3 and mRNA were significantly and inversely correlated (H3K9me3‐Up versus RNA‐Down and vice versa) in AD patients. The number of up‐ (red) or down‐regulated (green) genes involved in each GOBP is shown. (f) A representative down‐regulated (*RIMS1*) gene with increased H3K9me3 levels in AD patients (A1‐6) compared to normal control subjects (C1‐6). (g) A representative up‐regulated (*PTBP1*) gene with decreased H3K9me3 levels in AD patients (A1‐6) compared to normal control subjects (C1‐6). For each gene, read counts were displayed along exons and introns shown in the bottom. Relative normalized read counts were shown in the barplots. Significantly different at *p < .05; **p < .01

We performed mRNA‐seq on the same samples used for H3K9me3‐ChIP‐seq to investigate the relationship between AD‐related H3K9me3 changes and changes in the expression of target genes. On average, 67.8 million reads were obtained from individual samples and aligned to the human genome using TopHat (version 2.0.7), resulting in 5.5 Giga bps of the mapped sequences, which corresponds to 75.2‐fold coverage of the annotated human transcriptome (Table S6). We then identified 3,367 differentially expressed genes (DEGs; 1913 up‐regulated and 1,454 down‐regulated genes) as described in Section 4 (Table S7). To understand cellular processes associated with these AD‐related DEGs, we identified GOBPs represented by the 1913 up‐ and 1,454 down‐regulated genes using DAVID software (Figures 3a and 4c, Table S8). The down‐regulated genes were mainly involved in processes related to synaptic transmission (transmission of nerve impulse, neurotransmitter transport, neuron differentiation, and G protein‐coupled receptor signaling pathway). Up‐regulated genes were mainly involved in the processes related to cell adhesion, apoptosis, and metabolism. Differential expression for the two representative genes involved in amyloid precursor protein metabolic process and synaptic transmission, *KLK6* and *DLG4*, is shown in Figure 3b,c. While the expression level of *DGL4* (*p* < .01) was robustly reduced in AD, the expression of *KLK6* (*p* < .05) was moderately down‐regulated in AD. *DGL4* gene encodes postsynaptic density‐95 (PSD‐95), a postsynaptic scaffolding protein with multiple protein–protein interaction domains that is reduced in AD (Proctor, Coulson, & Dodd, 2010). Since *KLK6* gene is known to be expressed in endothelial cells of the brain, the increased RNA level of *KLK6* in AD may be due to elevated transcription in non‐neuronal cells. Currently, we could not unveil a mechanism on how the chromatin remodeling by H3K9me3 anticipates in the differential gene regulation in terms of repression versus depression. Based on our data, we propose that H3K9me3‐enriched chromatin orchestrates gene expression in a cell‐type‐specific manner and in a context‐dependent manner. A further study on the specific regulation of *DGL4* and *KLK6* expression remains to be investigated.

### 2.4 Integrated analysis of genome‐wide ChIP‐ and mRNA‐sequencing shows alterations of H3K9me3‐enriched epigenome signatures in AD

To define the link between enrichment of H3K9me3 and levels of mRNA expression in AD, we performed an integrated platform analysis and a stratified association analysis comparing H3K9me3‐enriched gene profiles between groups of the genes with high and low mRNA expression levels (Figure 4a,b, Figure S2). The comparison revealed that highly expressed genes showed decreased H3K9me3 levels in both promoters and gene bodies, compared to lowly expressed genes (Figure 4b). Importantly, they showed further decreases of H3K9me3 levels near the TSS. These decreases can be responsible for high expression of these genes, suggesting an inverse relationship between the levels of H3K9me3 and mRNA expression in AD. An independent correlation analysis further confirmed the inverse relationship between H3K9me3 and mRNA expression levels (Figure S3). Thus, among the DMGs and DEGs identified above, we focused on 90 genes showing the divergent changes in levels of mRNA expression and H3K9me3 (Figure S4c): (a) 46 genes with increased mRNA expression levels and decreased H3K9me3 levels and (b) 44 genes with decreased mRNA expression levels and increased H3K9me3 levels (Figure 4c,d). We then identified GOBPs represented by these genes by performing GOBP enrichment analysis using DAVID software (Figure 4e). These genes were mainly involved in the processes related to synaptic transmission (neuron projection morphogenesis, vesicle mediated transport, and GABA signaling), which were represented by the down‐regulated genes in AD (Figure 4e). Thus, these data suggest that the increase in the occupancy of H3K9me3 can be responsible for the down‐regulation of the genes involved in synaptic transmission. The representative gene with increased H3K9me3 levels in the promoters or gene bodies in AD was shown in Figure 4f,G.

### 2.5 Network analysis shows that H3K9me3‐landscaped epigenome signatures are associated with synaptic pathology of AD

**FIGURE 5:**
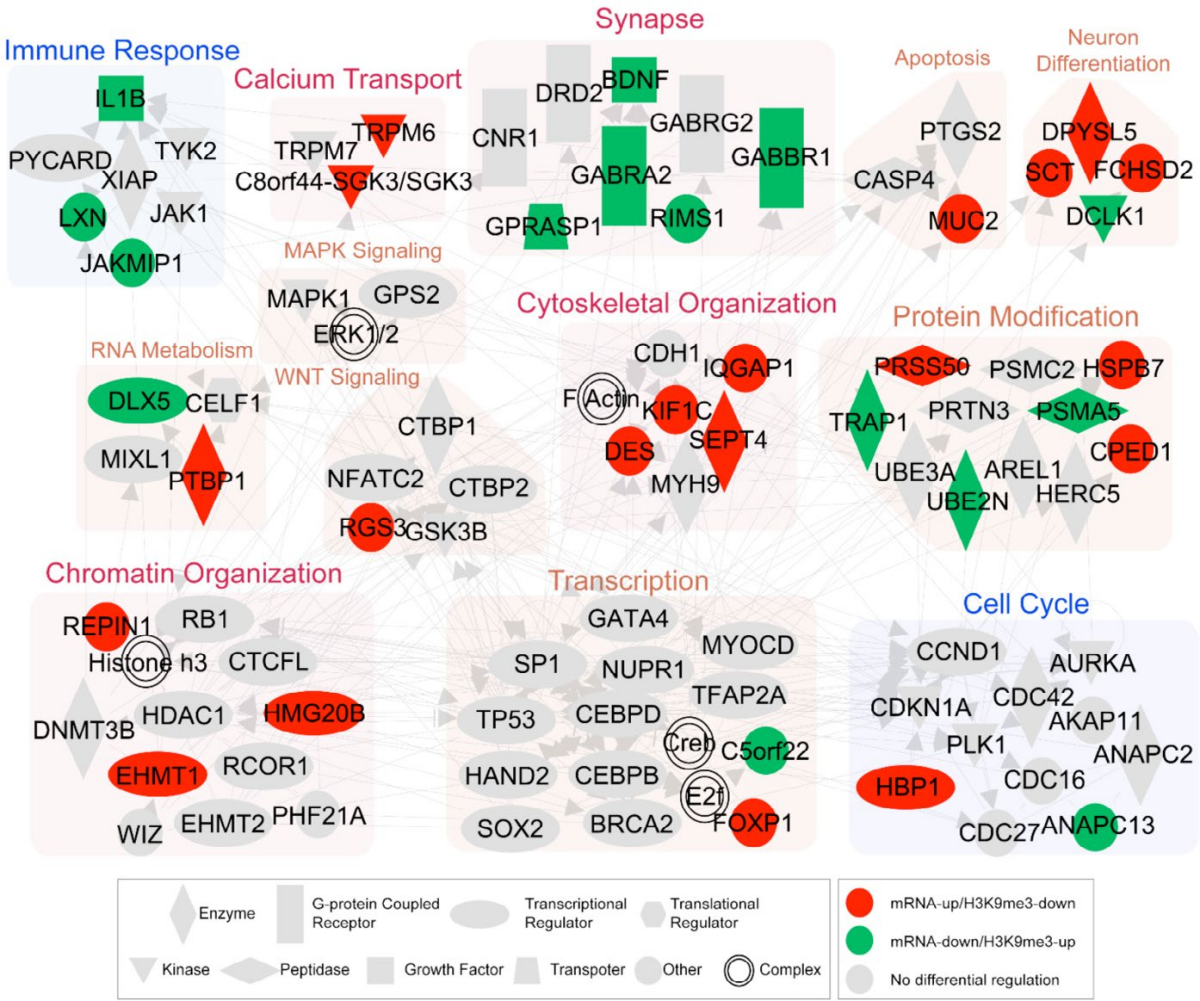
A network model shows that alteration of H3K9me3‐landscaped epigenome is linked to a systematic deregulation of AD‐related processes. Green nodes indicate the genes with decreased mRNA expression levels and increased H3K9me3 levels, whereas red nodes indicate the genes with increased mRNA expression levels and decreased H3K9me3 levels. Backgrounds represent the network modules for GOBPs in which the epigenomes in the modules are involved. Node shapes represent types of molecules as indicated in the legend. Gray nodes denote the genes added to connect the genes with the opposite changes of H3K9me3 and mRNA expression levels

To understand the functional associations of the H3K9me3‐landscaped epigenome in AD at the molecular level, we next generated a network model showing the interactions among the genes (DMGs and DEGs) with an inverse relationship between mRNA expression and H3K9me3 levels using IPA. The network showed dense connections among the genes associated with critical processes including calcium transport, synaptic transmission, and transcription regulation (Figure 5). Signaling molecules, neurotrophin (*BDNF*), WNT (*RGS3*), and calcium signaling‐related molecules (*TRPM6* and *C8orf44‐SGK3/SGK3*) closely interacted with the molecules involved in synaptic transmission (*GABRA2, GABBR1, GPRASP1*, and *RIMS1*), chromatin organization (*REPIN1, EHMT1,* and *HMG20B*), cell cycle (*HBP1* and *ANAPC13*), and transcription (*FOXP1* and *C5orf22*). The molecules involved in actin cytoskeleton reorganization (*DES, SEPT4, KIF1C,* and *IQGAP1*) mediated the interactions among the molecules in these processes. These data suggest that altered H3K9me3 levels may contribute to the pathogenesis of AD by modulating the expression of several genes involved in essential cellular processes.

### 2.6 Quantitative real‐time PCR (qPCR) verifies that H3K9me3‐lanscaped epigenomes are differentially regulated in AD

**FIGURE 6:**
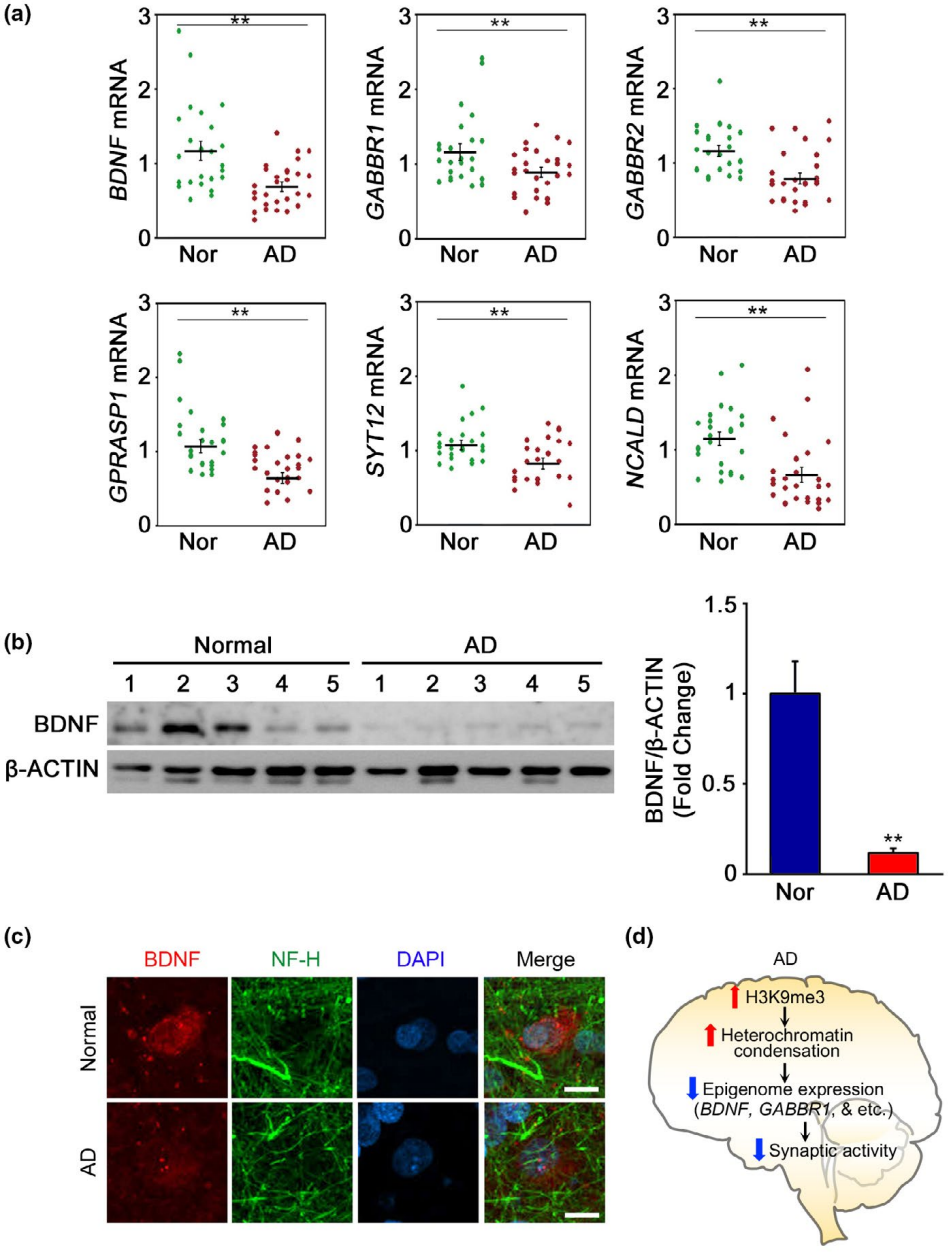
H3K9me3‐landscaped and synaptic function‐related transcriptome are deregulated in AD. (a) The expression of *BDNF*, *GABBR1*, *GABRA2*, *GPRASP1*, *SYT12*, and *NCALD* were down‐regulated in AD patients (*N* = 28) compared to normal control subjects (*N* = 24). Significantly different at *p < .05; **p < .001. (b) The protein levels of BDNF were reduced in AD patients (*N* = 5) compared to normal control subjects (*N* = 5). (c) BDNF immunoreactivity is reduced in neurofilament heavy chain (NF‐H)‐positive cortical neuron of AD patient. Scale bars (white): 5 µm. (d) A scheme showing that increased H3K9me3 levels and heterochromatin condensation contribute to synaptic deregulation in AD by repressing the expression of synaptic function‐related genes (such as *BDNF* and *GABBR1*)

To further verify the alterations of the H3K9me3‐landscaped transcriptome in AD, we performed qPCR and confirmed target gene expression (mRNA levels) in the temporal cortex of normal and AD brains. qPCR confirmed alterations of the brain‐derived neurotrophic factor (*BDNF*), GABA B receptor 1 (*GABBR1*), GABA A receptor 2 *(GABRA2*), G protein‐coupled receptor‐associated sorting protein 1 (*GPRASP1*), synaptotagmin XII (*SYT12*), neurocalcin Delta (*NCALD*), septin 4 (*SEPT4*), kinesin family member 1C (*KIF1C*), inhibitor of DNA binding 3 (*ID3*: dominant helix‐loop‐helix protein), and histone cluster 2, H2be (*HIST2H2BE*) (Figure 6a, Figure S4a,b). mRNA levels of the following genes in which H3K9me3 promoter occupancy was increased were significantly reduced in AD: *BDNF*, *GABBR1*, *GABRA2*, *GPRASP1*, *SYT12*, and *NCALD* (Figure 6a). BDNF protein level and immunoreactivity were significantly reduced in cortical neurons of AD patients (Figure 6b,c). In contrast, *SEPT4* and *KIF1C* genes, in which H3K9me3 promoter occupancy is decreased, and *ID3* and *HIST2H2BE* genes, in which H3K9me3 promoter occupancy is increased, all exhibited increased mRNA levels (Figure S4a,b). Figure 6d summarizes how increased H3K9me3 levels and heterochromatin condensation repress synaptic function‐related genes (such as *BDNF* and *GABBR1*) in AD.

### 2.7 Brain cell‐type‐specific H3K9me3‐enriched epigenome profiling

We ran an integrative analysis to compare the number of DMGs and DEGs (H3K9me3‐landscaped epigenome) in AD (Figure S5a, Tables S4 and S7). And we selected 182 common epigenomes merged from 1,388 DMGs and from 3,366 DEGs. To identify biological functions associated with AD, we conducted GO gene set enrichment analysis (GSEA) for 182 common epigenomes (Figure S5b). Notably, a neuron part pathway was most significantly affected among top 10 GO pathways in AD. In order to sort brain cell‐type‐specific H3K9me3‐landscaped epigenomes, we designated 5 cell types including neurons, astrocytes, oligodendrocytes, microglia, and endothelial cells from human brain single cell RNA‐sequencing dataset (Darmanis et al., 2015) (Figures S5–S8, Table S4 and S7). In neurons, 5 epigenomes (*FCHSD2, SEPT4, KIF1C*, *KLHL17,* and *DPYSL5*) were up‐regulated in AD (Figure S5c). We also identified 8 down‐regulated genes including *DCLK1, RBM3, GABRA2, BDNF, SYT12, RIMS1, LAMP5,* and *DLGAP2* in neurons (Figure S5d). In astrocytes, we found 2 common genes such as *SLC25A48* and *LIFR* from 13 DMGs and from 26 DEGs (Figure S8a). The *SLC25A48* epigenome was up‐regulated in astrocytes (Figure S8b). In oligodendrocytes, *SERTAD3* was up‐regulated in AD (Figure S8b). In microglia, *IL1B* epigenome was down‐regulated in AD (Figure S8c). In endothelial cells, *KLF10, ABCG2, SLC39A8, ETS1,* and *ZC3HAV1*genes were landscaped by H3K9me3 but there were no common genes overlapped between DMGs and DEGs (Figures S6d and S8).

## 3 DISCUSSION

Dynamic changes in chromatin structure are a prominent pathological feature of many neurodegenerative diseases (Houston et al., 2013; Jakovcevski & Akbarian, 2012; Lee et al., 2013; Maze et al., 2011). In general, acetylation of lysine residues in the N‐terminal of histone molecules corresponds to transcriptionally active chromatin (euchromatin) that promotes transcription. In contrast, methylation of certain lysine residues contributes to transcriptionally inactive chromatin (heterochromatin) condensation and represses transcription or vice versa (Sadri‐Vakili & Cha, 2006). The post‐translational modifications of histone proteins in AD likely promote alterations in chromatin packaging that may affect the expression of neuronal genes (Ryu et al., 2008). Because H3K9me3 is highly enriched in the intergenic regions of genome such as pericentromeres and gene deserts, H3K9me3 is thought to be associated with the maintenance of heterochromatin structure (Lee et al., 2013; Maze et al., 2011; Rosenfeld, Xuan, & DeSalle, 2009). In addition, histone H3K9 methylation is associated with decreased transcriptional activity as a repressive “histone code” in contrast to histone H3K9 acetylation that increases transcription (Hake, Xiao, & Allis, 2004; Wu et al., 2007). Nevertheless, the alteration of histone H3K9 methylation and the expression of the H3K9me3‐landscaped epigenome have not been fully investigated in AD. In the present study, we show that the level of histone H3K9me3 is apparently altered in AD confirming our previous preliminary observations (Ryu et al., 2008).

In the present study, we found that H3K9me3‐positive heterochromatin condensation was elevated throughout many regions of the cortex in APP/PS1 mutant mice (Figure S9). Moreover, H3K9me3‐postive chromatin condensation was robustly and broadly increased throughout many regions of the cortex in 5xFAD mice. Our finding on the elevation of H3K9me3‐positive heterochromatin condensation in AD is concurrent with previous studies using AD monozygote twins and AD transgenic mouse models (Walker, LaFerla, Oddo, & Brewer, 2013). Otherwise, previous reports show that tau‐mediated toxic effect leads to H3K9me2‐positive heterochromatin relaxation and loss in a fly model of AD (Frost, Bardai, & Feany, 2016; Frost, Hemberg, Lewis, & Feany, 2014). Hajjar et al. (2019) also present that H3K9me3‐assoicated heterochromatin relaxation is found in *Bmi1* ^+/−^ mice similar to AD (El Hajjar et al., 2019). In general, H3K9me1 and H3K9me2 are accumulated at perinuclear chromatin foci while H3K9me3 is condensed in pericentromeric foci via H3K9me3‐specific methyltransferase (Town et al., 2012). Based on the previous reports and our current findings, we propose that the H3K9me3‐specific heterochromatin condensation is observed in structurally compact nuclei of neuronal cells while the heterochromatin relaxation or loss can be found in structurally disrupted nuclei of neuronal cells under degenerative stresses (Hajjar et al., 2019; Frost et al., 2014, 2016; El Town et al., 2012; Walker et al., 2013). Together, the difference of heterochromatin dynamics and structures may be observed dependent upon the severity of AD pathology, the brain region, and the experimental animal model (Hajjar et al., 2019; Frost et al., 2014, 2016; El Town et al., 2012; Walker et al., 2013). We consider that one AD animal model cannot precisely replicate or mimic AD pathology in patients. Accordingly, an exact mechanism on how heterochromatin condensation and relaxation is modulated under neurodegenerative conditions remains to be further investigated in more AD postmortem specimens and animal models in future studies.

We performed an integrated analysis of H3K9me ChIP‐sequencing and mRNA‐sequencing data and identified 90 genes showing inverse relationships between the changes of H3K9me3 and mRNA expression levels in AD. Importantly, repressed gene profiles by H3K9me3 mark were mainly involved in synaptic transmission. The network analysis further showed dense interactions among genes involved in synaptic transmission, neuronal differentiation, and cell motility. This analysis illustrates that the alteration of H3K9me3‐landscaped epigenomes systematically contributes to the deregulation of neuronal processes that are found in AD. While H3K9me3 occupancy is increased within the promoters of *BDNF, GABBR1, GABRA2,* and *GPRASP1*, the mRNA levels of these genes were significantly decreased in AD. Since synapses are the primary target of neuronal damage, the progressive synaptic dysfunction is closely linked to cognitive deterioration in AD. BDNF is one of the key regulators of synaptic plasticity and memory consolidation, and it is deficient in AD brains (Lee et al., 2012; Raiha et al., 1998; Ravaglia et al., 2005; Su et al., 2004). However, the mechanisms regulating the expression of *BDNF* in AD are poorly understood. It has been known that *BDNF* expression is modulated epigenetically by miRNAs in the brain (Lee et al., 2012). In the current study, we further identified that H3K9me3 down‐regulates *BDNF* expression through the modulation of chromatin remodeling in the *BDNF* promoter region in AD. *SYT* and *GABRA2* are known to be decreased in AD postmortem brains (Gebhardt, Scott, & Dodd, 2010; Siegmund et al., 2007). Our current data show that H3K9me3 is enriched in the promoter region of *GABBR1* and *GABRA2* genes and that repressive marks are associated with the repression of these genes. Expression of the *SEPT4* gene, which encodes the Drosophila orthologue of human CDCrel‐1, was elevated in AD due to the chromatin remodeling by H3K9me3. SEPT4 is known to be a Parkin substrate and a Lewy body protein that is found in the brains of PD patients (Shehadeh, Mitsi, Adi, Bishopric, & Papapetropoulos, 2009). Ectopic expression of SEPT4 results in age‐dependent disruption of DA neuron integrity, and its negative role is antagonized by Parkin. The increase of *SEPT4* expression suggests that accumulation of SEPT4 may be toxic for neurons that are associated with memory function in AD, but the exact role for SEPT4 remains to be further investigated.

We also found that *ID3* gene expression is elevated in AD brain. ID3 is a helix‐loop‐helix transcriptional regulator that plays a novel role in neurogenesis and cell cycle progression (Kee & Bronner‐Fraser, 2005; Lyden et al., 1999; Riechmann, van Crüchten, & Sablitzky, 1994). Despite ID subfamily genes (ID1, ID2, ID3, and ID4) have been identified as epigenetic targets in the regulation of neuronal maturation and the molecular pathogenesis of Rett syndrome (RTT), the role of ID3 in neurodegenerative conditions and in the pathogenesis of AD is not known yet (Peddada, Yasui, & LaSalle, 2006). Considering that the cell cycle entry mechanism is associated with neuronal damage in AD and that the expression level of *ID3* is increased in AD, we propose that cell cycle progression triggered by ID3 may lead to a conflict of neuronal fate to proliferate or to differentiate, in turn, which may cause neuronal damage. Further functional studies may warrant for elucidating the exact roles of these candidate genes in the pathogenesis of AD.

Transcriptional anomalies are observed in AD, in which a subset of genes identified by expression profiling is significantly dysregulated (Robakis & Georgakopoulos, 2014). Indeed, altered gene transcription in AD has been associated with alterations in histone acetylation profiles (Chouliaras et al., 2010; Kilgore et al., 2010). In both cases, neuropathogenic alterations of transcriptional activity lead to perturbations in normal neuronal function, resulting in neuronal damage. Epigenetic modifications can explain the synaptic dysfunction and the pathogenesis in AD (Lee & Ryu, 2010; Maloney & Lahiri, 2016). For example, histone modifications, DNA methylation, ribosomal DNAs (rDNAs), and microRNAs (miRNAs) have been suggested as epigenetic factors mediating the influence of environmental factors on AD‐related gene expression (Gräff et al., 2012; Mastroeni et al., 2010; Daniel et al., 2014; Zeng, Libien, Shaik, Wolk, & Hernández, 2016). Interestingly, histone deacetylase 2 (HDAC2) is increased within the hippocampus of AD patients, indicating that histone deacetylation is associated with the AD (Gräff et al., 2012). Moreover, DNA (cytosine‐5) methyltransferase 1 (DNMT1) is significantly reduced in neurons of entorhinal cortex in AD patients (Mastroeni et al., 2010). It seems likely that various environmental stresses influence chromatin remodeling and gene expression that advances and leads to the onset as well as the disease progression of AD. It has been suggested that gradual epigenetic changes are more upstream in AD pathogenesis than the more conventional pathological phenotypes such as Aβ‐dependent senile plaque formation and tauopathy (Adwan & Zawia, 2013). Together, abnormal epigenetic alterations may be causative to induce gradual impaired cognitive functions and pathogenesis of AD (Maloney & Lahiri, 2016). Consistent with this notion, our findings indicate that it is possible that neuronal genes may become inactive or active through the engagement of H3K9me3 mark as neurodegeneration proceeds in response to environmental stresses. As AD progresses, the reversible decondensation of H3K9me3‐enriched heterochromatin may be impaired and lead to the constitutive down‐regulation of synaptic gene expression. This pathological alteration of epigenetic signaling pathways may contribute to the progressive and irreversible nature of neurodegeneration in AD. We have previously reported epigenetic alteration in a monozygote twin with discordance AD (Ryu et al., 2008). In this case, a monozygote twin with AD shows an increase of H3K9me3 level and heterochromatin condensation in comparison with a normal monozygote twin. These data implicate that H3K9me3‐dependent epigenetic modification may explain another layer of plausible mechanism beyond genetic‐perspective view of AD pathogenesis. Otherwise, in familial AD animal models (APP/PS1 mutant and 5xFAD mouse model), we found an increase of H3K9me3‐dependent heterochromatin condensation (Figure S9). Together, we propose that epigenetic alterations may be pivotal pathological features in both sporadic and familial AD cases. However, it will be necessary to further investigate what epigenome signatures are differently expressed in sporadic versus familial AD cases in a future study. The mechanisms responsible for H3K9me3 elevation in AD and whether H3K9me3‐landscaped chromatin structures and genes are reversibly modulated are important topics for future studies.

In summary, our integrated analysis of H3K9me3‐ChIP‐sequencing and RNA‐sequencing data disclosed changes affecting a list of epigenomes involved in synaptic transmission, neuronal differentiation, and cell motility in AD. Our findings suggest that H3K9me3 is intimately involved in the systematic epigenetic alterations found in AD and that H3K9me3‐landscaped genes are associated with AD pathogenesis.

## 4 MATERIALS AND METHODS

A detailed description of the materials and methods (Cell‐type‐specific designation analysis, Chromatin immunoprecipitation (ChIP), ChIP‐sequencing, mRNA‐sequencing, Analysis of mRNA‐seq data, Quantitative real‐time PCR (qPCR), Western blot analysis, and Confocal microscopy) is available in Supporting Information.

### 4.1 Neuropathological diagnosis

A total of 40 autopsy participants were examined from Boston University's Alzheimer's Disease Center (BUADC) with and without cognitive impairment who underwent annual cognitive evaluations using the National Alzheimer's Disease Coordinating Center (NACC) Uniform Data Set (UDS) protocol (Beekly et al., 2007). Consents for brain donation and research participation were provided by the donor's next of kin. Institutional review boards from the Boston University Medical Center approved brain donation, postmortem clinical record review, neuropathological evaluation, and clinical interviews with donor family members. Neuropathological assessment was performed by following procedures and criteria as previously established (Mez et al., 2015; Vonsattel, Amaya, Cortes, Mancevska, & Keller, 2008). The neuropathological diagnosis for Alzheimer's disease was performed by board‐certified neuropathologists (T.D.S. and A.C.M.) based on the National Institute of Aging Reagan criteria and included intermediate or high probability (Montine et al., 2012). Only brain tissues with the Braak Stage of I to II were used for ChIP‐ and RNA‐Seq in normal subjects. In cases of AD patients, the Braak Stage of V to VI were used for ChIP‐ and RNA‐Seq. Detailed information of brain tissues is described in Table S1.

### 4.2 Analysis of ChIP‐sequencing data

Adapter sequences and bases with quality scores less than 20 were trimmed using cutadapt (Martin, 2011). All reads were mapped to the human reference genome (GRCh37, version 19) using bowtie (version 2.0.6) with default settings (Langmead & Salzberg, 2012). Duplicated reads at the same genomic location were discarded using Picard [http://broadinstitute.github.io/picard/]. The mapped reads with MAPQ <5 were discarded. To identify the genes with changes in H3K9me3 levels between AD and normal samples, we used the following two methods. In the first method, we identified the peaks of H3K9me3 using MACS (version 1.4.2) with the default parameters (Zhang et al., 2008). Identified peaks were aligned across the samples, and the consensus peak regions were defined using DiffBind (Ross‐Innes et al., 2012). Only the peaks identified in two or more samples were used. For each sample, read counts were calculated in the consensus regions and normalized by the trimmed mean of M‐values (TMM) method (Robinson & Oshlack, 2010). Using the normalized read counts, differentially marked regions (DMRs) were then identified by edgeR as the ones with *p* < .05 and fold‐changes > a cutoff, which was determined as 10th percentile of the fold‐change distribution obtained by performing random permutation experiments 1,000 times (Robinson, McCarthy, & Smyth, 2010). Finally, 66 genes including the DMRs were identified as differentially marked genes (DMGs). In the second method, we compared the total read counts in promoter and gene body regions between AD and normal samples. For each gene, the promoter region was defined from the transcription start site (TSS) to 3kb upstream, and the gene body was defined from TSS to transcription end site (TES). The same TMM method was used for normalization of the read counts, and edgeR was then used to identify the DMGs in the promoter and gene body (1,111 and 224 DMGs, respectively). Finally, we combined the two sets of the DMGs (1,388 DMGs) identified from the two methods and used them for the following analyses.

### 4.3 Expression stratified association analysis of H3K9me3 profiles in the gene structure

The average mRNA expression levels of all the samples were sorted, and the two sets of the 1,000 genes with the largest (High) and smallest (Low) average mRNA expression levels, respectively, were selected. The genes with the lengths <5 Kbp were not included in the selected gene sets. For each gene in the High set, the gene structure spanned from 5 kb upstream of transcription start site (TSS) to 5 kb downstream of transcription end site (TES), and the whole region was divided into 100 bins. The mean read counts of the 1,000 genes in the High set were calculated in individual bins using ngsplot (Shen, Shao, Liu, & Nestler, 2014). The same procedure was used for the genes in the Low set.

### 4.4 Functional enrichment analysis

To identify cellular processes represented by a list of genes, we performed functional enrichment analysis of GO biological processes (GOBPs) and Kyoto Encyclopedia of Genes and Genomes (KEGG) pathways using DAVID software and selected the GOBPs represented by the genes as the ones with *p* < .1 (default cutoff) in DAVID.

### 4.5 Network analysis

We generated a network model to show the interactions among the genes showing the anti‐correlated changes of mRNA expression and H3K9me3 levels using Ingenuity Pathway Analysis (IPA; QIAGEN). Only the interactions with experimental evidence between the genes were used. We first used the network generation algorithm in IPA to generate the subnetworks for a set of the genes and then merged the interconnected subnetworks into a single network model. In the network model, we grouped the nodes with the similar GOBPs or in the same KEGG pathways into the same modules, each of which was named by the corresponding GOBP or KEGG pathway.

### 4.6 Statistical analysis

The qPCR data and densitometry data from Western blot are presented as the mean ± *SE*. The data analysis between normal and AD group was performed by Student's *t* test and one‐way ANOVA followed by Fisher's protected least significant difference test using StatView 4 (Abacus Concepts). Differences were considered statistically significant at *p* < .05.

## CONFLICT OF INTEREST

The authors declare no competing financial interests.

## AUTHOR CONTRIBUTIONS

J.‐I.K., D.H., and H.R. contributed to conceptualization and design of the study. A.C.M. and T.D.S. contributed to human postmortem brain resources. M.Y.L., H.C., J.‐Y.S., and D.H. contributed to RNA‐sequencing and ChIP‐sequencing data analysis. Y.J.H. and S.J.H. contributed to immunohistochemistry, Western blot analysis, and other experiments. M.Y.L., D.H., J.L., and H.R. contributed to writing—original draft. N.W.K. contributed to writing—review and editing.

## Supporting information

Supplementary Material

Table S4

Table S7

## Data Availability

The sequencing data generated in this study were deposited to European Nucleotide Archive (ENA), and the following accession number has been assigned: PRJEB36676.
