# Supplementary material for: Epigenome signatures landscaped by histone H3K9me3 are associated with the synaptic dysfunction in Alzheimer's disease
Source: Aging Cell. 2020 May 17;19(6):e13153. doi: 10.1111/acel.13153 (PMC7294781; doi:10.1111/acel.13153)
Supplement: Supplementary file 1 — Supplementary Material [file ACEL-19-e13153-s001.docx]

**Supplementary Information**

**Title:**

**Epigenome signatures landscaped by histone H3K9me3 are associated with the synaptic dysfunction in Alzheimer's disease**

Min Young Lee, Junghee Lee, Seung Jae Hyeon, Hyesun Cho, Yu Jin Hwang,

Jong-Yeon Shin, Thor Stein, Ann C. McKee, Neil W. Kowall, Jong Il Kim*,*

Daehee Hwang, and Hoon Ryu

**Supplementary Methods**

***Brain cell-type-specific designation analysis***

The common genes that are landscaped by H3K9me3 were analyzed by the Molecular Signatures Database (MSiqDB) of Gene Set Enrichment Analysis (GSEA) from DMGs and DEGs [2]. We selected the Kyoto Encyclopedia of Genes and Genomes (KEGG) gene set of MSiqDB. Hierarchical clustering was performed using average linkage method of Cluster 3.0 [3].

Brain cell-type (neurons, astrocytes, oligodendrocytes, endothelial and microglia)-specific genes were computed and sorted by designating a large scale human brain single-cell RNA seq dataset [1]. Cell-type-specific genes were filtered by several criteria [4]. The filtered cell type-specific genes (1032 neurons, 191 astrocytes, 111 oligodendrocytes, 76 endothelial, 118 microglia) were sorted based on H3K9me3 occupancy (DMGs) and gene expression (DEGs).

***Chromatin immunoprecipitation (ChIP)***

ChIP for H3K9me3-DNA was performed using a CHIP assay kit (Santa Cruz) as described previously [15]. Brain tissues collected from AD patients and normal subjects were cross-linked with 1 % formaldehyde for 20 min at room temperature. The lysates were sonicated six times with each time for 30 s using Bioruptor (Diagenode Inc., NJ, USA). After centrifugation, the supernatant was diluted in CHIP dilution buffer and then incubated overnight at 4 °C with anti-H3K9me3 antibody. Immune complexes were recovered by the addition of 60 μl of salmon sperm DNA/protein A agarose-50 % slurry and incubation for 2h at 4 °C with rotation. The beads were pelleted and washed with low and high salt buffer and finally three times with LiCl buffer. The immune complexes were eluted by incubation for 4 h at 66 °C with 500 μl of fresh elution buffer (1 % SDS, 0.1 M NaHCO3) and 20 μl of 5 M NaCl. The DNA cleaned by the addition of 2 μl of 500 mM EDTA, 1 μl of 1 M Tris and 1 μl of proteinase K. The DNA solution is extracted with a phenol/chloroform/isoamyl alcohol mixture to remove protein contaminants, then precipitated with 100 % ethanol. After the precipitation step, the pellet was washed with 70 % ethanol and dissolved in 20 μl DW.

***Quantitative real-time PCR (qPCR)***

Fifty nanograms of RNA were used as a template for qPCR amplification, using SYBR Green Real-time PCR Master Mix (Toyobo, Japan) as previously described. Two-step PCR thermal cycling for DNA amplification and real-time data acquisition was performed with an ABI StepOnePlus^TM^ Real-Time PCR System using the following cycle conditions: 95 °C for 1 min × 1 cycle, and 95 °C for 15 s, followed by 60 °C for 1 min × 40 cycles. Fluorescence data were analyzed by the ABI StepOnePlus software and expressed as *C*_T_, the number of cycles needed to generate a fluorescent signal above a predefined threshold. The ABI StepOnePlus software set baseline and threshold values. Primers were standardized in the linear range of cycles prior to onset of the plateau. The mRNA was normalized to GAPDH. The qPCR primer sequences that are used in this study, is described in Supplementary Table 9.

***ChIP-sequencing***

ChIP-seq library were constructed from the ChIP DNA samples following Illumina’s Chip-seq sample preparation protocol. Briefly, Chip DNA was end-blunted and added with an ‘A’ base such that the adaptors from Illumina with a ‘T’ could ligate on the ends. 200–400 bp fragments were then gel-isolated and purified. The library was amplified by 18 cycles of PCR. The resulting DNA libraries were quantified by an Agilent DNA 1000 series II assay and a Nanodrop 7500 spectrophotometer using a 1.5 μl aliquot diluted to 10 nM. We performed cluster generation and 36 cycles of sequencing on the Illumina cluster station and HiSeq2000 according to the manufacturer’s instructions. Basic analyses of ChIP-seq data, such as image analysis and base calling, were performed using the Illumina’s Genome Analysis pipeline.

***mRNA-sequencing***

We first prepared samples for mRNA-seq following the Illumina standard protocol. Briefly, 3 μg of total RNA from each sample was used for polyA mRNA selection using streptavidin-coated magnetic beads, followed by thermal mRNA fragmentation. The fragmented mRNA was reverse-transcribed to generate cDNA using reverse transcriptase (SuperScript II) and random primers, which was further converted into double-stranded cDNA. After an end repair process (Klenow fragment, T4 polynucleotide kinase and T4 polymerase), the resulting cDNA was finally ligated to Illumina paired end (PE) adaptors. Using a 2 % agarose gel, cDNA libraries ranging in size between 200 and 250 bp were selected, subjected to ten cycles of PCR, and then purified using the QIAquick PCR purification kit (Qiagen). The enriched libraries were diluted with Elution Buffer to a final concentration of 10 nM. Finally, 8 pM of the library in each sample was sequenced using the HiSeq2000 with 101 bp sequencing.

***Analysis of mRNA-seq data***

Adapter sequences and bases with quality scores less than 20 were trimmed using cutadapt [16]. All reads were mapped to the human reference genome (GRCh37, version 19) using TopHat (version 2.0.7,) with the default parameters [23]. Duplicated reads at the same genomic location were discarded using Picard, and the mapped reads with MAPQ<5 were discarded [18]. For each gene, the number of reads in the exons were counted using R package GenomicFeatures [24]. The genes with zero counts across all samples were discarded. The read counts were normalized by the TMM method [21]. We first identified expressed genes as the ones having read counts larger than 5, which corresponded to 0.2 count per million (CPM) and log_2_CPM = -2.32 in our datasets, according to the instruction in the user’s guide of edgeR package [25]. Then, the differentially expressed genes (DEGs) were identified from the expressed genes with log_2_CPM > -2 using the two different methods, EdgeR and DESeq [22, 26]. Each method computed the significance (P-value) of a gene being differentially expressed. In each method, the DEGs were selected as the genes with P < 0.05 and fold-changes > a cutoff, which was determined as a minimal fold-change cutoff, the 10^th^ percentile (1.38-fold) of the fold-change distribution obtained by performing random permutation experiments 1000 times, as previously described [27, 28]. Finally, we combined the two sets of the DEGs identified from the two methods (3290 and 1483 DEGs from EdgeR and DESeq, respectively) and used them for the following analyses.

***Western blot analysis***

Western blot was performed as previously described [12]. Thirty microgram of protein was subjected to SDS-PAGE (10%) and blotted with anti-H3K9me3 (Upstate Biotech.), anti-ESET (Santa Cruz Biotech.) antibody. Protein loading was controlled by probing for histone H3 (Upstate Biotech) or alpha-tubulin (Sigma) on the same membrane.

***Confocal microscopy***

The tissue sections were rehydrated, blocked with blocking solution (5% BSA), and incubated with anti-H3K9me3 antibody (1:200 dilutions) (Upstate Biotech.) for 24 hr as previously described [15]. After three times of washing, the slides were incubated with Cy3-conjugtared secondary antibody. The nuclei were counterstained with DAPI (4',6-diamidino-2-phenylindole). Images were analyzed using a Spinning Disk Confocal microscope (IX2-DSU, Olympus). Preabsorbtion with excess target protein or omission of primary antibody was used to demonstrate antibody specificity and background generated from the detection assay.

**Supplementary References**

1. Darmanis, S. et al., A survey of human brain transcriptome diversity at the single cell level. *Proc Natl Acad Sci U S A*, 2015. **112**:7285-7290.

2. Subramanian, A. et al., Gene set enrichment analysis: a knowledge-based approach for interpreting genome-wide expression profiles. *Proc Natl Acad Sci U S A*, 2005. **102**:15545-15550.

3. de Hoon, M.J. et al., Open source clustering software*.* *Bioinformatics*, 2004. **20**: 1453-1454.

4. Wang, M. et al., Integrative network analysis of nineteen brain regions identifies molecular signatures and networks underlying selective regional vulnerability to Alzheimer's disease*.* *Genome Med*, 2016. **8**:104.

**Supplementary Table 1. Information on brain tissue samples from normal subjects and AD patients.**

| Number | Case | Age | Sex | Braak stage |
| --- | --- | --- | --- | --- |
| 1 | Normal | 87 | Female | I |
| 2 | Normal | 88 | Male | I |
| 3 | Normal | 86 | Male | II |
| 4 | Normal | 87 | Female | II |
| 5 | Normal | 67 | Male | I |
| 6 | Normal | 82 | Male | I |
| 7 | Normal | 61 | Male | I |
| 8 | Normal | 101 | Female | I |
| 9 | Normal | 89 | Male | III |
| 10 | Normal | 68 | Male | I |
| 11 | Normal | 78 | Female | I |
| 12 | Normal | 89 | Male | III |
| 13 | Normal | 70 | Male | I |
| 14 | Normal | 82 | Male | I |
| 15 | Normal | 79 | Female | I |
| 16 | Normal | 70 | Male | I |
| 17 | Normal | 59 | Male | II |
| 18 | Normal | 80 | Female | I |
| 19 | Normal | 92 | Male | I |
| 20 | Normal | 90 | Female | I |
| 21 | AD | 85 | Male | V |
| 22 | AD | 78 | Male | V |
| 23 | AD | 80 | Male | VI |
| 24 | AD | 70 | Female | VI |
| 25 | AD | 79 | Male | IV |
| 26 | AD | 87 | Female | V |
| 27 | AD | 82 | Male | V |
| 28 | AD | 79 | Female | VI |
| 29 | AD | 70 | Male | VI |
| 30 | AD | 59 | Male | VI |
| 31 | AD | 80 | Female | V |
| 32 | AD | 92 | Male | V |
| 33 | AD | 90 | Female | V |
| 34 | AD | 100 | Male | V |
| 35 | AD | 75 | Male | V |
| 36 | AD | 83 | Male | VI |
| 37 | AD | 79 | Female | VI |
| 38 | AD | 89 | Male | IV |
| 39 | AD | 69 | Male | VI |
| 40 | AD | 79 | Female | V |

**Supplementary Table 2. Summary of H3K9me3 ChIP-sequencing alignment. AD, Alzheimer’s disease patient; C, control normal subject.**

| **Case** | **Total reads** | **Filtered** | **Used** | **Aligned** | **Uniquely aligned reads** | **Alignment ratio** | **Depth (fold)** |
| --- | --- | --- | --- | --- | --- | --- | --- |
| AD1 | 49,822,698 | 4,186,387 | 45,636,311 | 24,964,206 | 17,534,648 | 0.55 | 0.51 |
| AD2 | 45,113,702 | 5,425,981 | 39,687,721 | 22,793,875 | 16,659,985 | 0.57 | 0.52 |
| AD3 | 53,280,572 | 5,698,574 | 47,581,998 | 36,671,192 | 28,280,368 | 0.77 | 0.89 |
| AD4 | 43,906,144 | 5,989,777 | 37,916,367 | 23,702,350 | 17,392,118 | 0.63 | 0.57 |
| AD5 | 83,255,608 | 6,717,509 | 76,538,099 | 69,711,657 | 57,005,146 | 0.91 | 2.00 |
| AD6 | 51,795,958 | 4,770,452 | 47,025,506 | 35,977,097 | 27,486,222 | 0.77 | 0.90 |
| C1 | 35,856,132 | 4,589,308 | 31,266,824 | 19,235,296 | 13,082,606 | 0.62 | 0.40 |
| C2 | 51,522,722 | 5,482,295 | 46,040,427 | 35,107,493 | 26,445,487 | 0.76 | 0.86 |
| C3 | 27,902,276 | 5,552,044 | 22,350,232 | 14,795,547 | 10,473,113 | 0.66 | 0.34 |
| C4 | 60,184,128 | 5,522,863 | 54,661,265 | 41,810,285 | 32,693,238 | 0.76 | 1.03 |
| C5 | 30,778,772 | 3,032,163 | 27,746,609 | 23,064,566 | 17,877,738 | 0.83 | 0.63 |
| C6 | 43,931,796 | 3,918,104 | 40,013,692 | 31,535,082 | 24,724,978 | 0.79 | 0.80 |

**Supplementary Table 3. H3K9me3 occupancy in the promoter, intergenic, and gene body regions. The gene body occupancy was further divided by CDS, UTR, and introns. AD, Alzheimer’s disease patient; C, control normal subject.**

| **Case** | **%**  **Promoter** | **% Intergenic** | **%**  **Gene body** | **%**  **CDS** | **%**  **UTR** | **%**  **Intron** |
| --- | --- | --- | --- | --- | --- | --- |
| AD1 | 2.27 | 62.76 | 34.97 | 2.43 | 2.63 | 94.94 |
| AD2 | 2.52 | 58.47 | 39.02 | 2.43 | 2.71 | 94.86 |
| AD3 | 2.63 | 57.63 | 39.74 | 2.42 | 2.80 | 94.77 |
| AD4 | 2.55 | 58.71 | 38.75 | 2.45 | 2.74 | 94.81 |
| AD5 | 2.90 | 54.42 | 42.68 | 2.48 | 2.90 | 94.61 |
| AD6 | 2.27 | 59.66 | 38.07 | 2.25 | 2.65 | 95.10 |
| C1 | 2.36 | 60.15 | 37.48 | 2.62 | 2.66 | 94.71 |
| C2 | 2.40 | 58.37 | 39.22 | 2.53 | 2.72 | 94.75 |
| C3 | 2.32 | 59.15 | 38.52 | 2.49 | 2.67 | 94.84 |
| C4 | 2.69 | 56.20 | 41.10 | 2.54 | 2.82 | 94.64 |
| C5 | 2.75 | 56.99 | 40.26 | 2.72 | 2.89 | 94.39 |
| C6 | 2.59 | 56.79 | 40.62 | 2.59 | 2.80 | 94.61 |

**Supplementary Table 4. DMGs identified by MACS and EdgeR. EdgeR-P and GB represent the DMGs whose promoters or gene bodies were differentially marked between the cortex of AD patients and normal subjects (Please confer an attached excel file including Supplementary Table 4 and 7).**

**Supplementary Table 5. GOBPs represented by the DMGs. 'Increased' and 'Decreased' represent the GOBPs represented by the genes with increased and decreased H3K9me3 occupancy in the promoter region of gene, respectively. 'Count' means the number of the genes involved in the corresponding GOBP. Such genes are listed.**

|  | **Term_ID** | **Description** | **Count** | **P-value** | **Genes** |
| --- | --- | --- | --- | --- | --- |
| **Increased** | GO:0050877 | neurological system process | 61 | 0.00 | OR1A2, LXN, MYO7A, OR11A1, OR11H12, KIFC3, CTNNB1, CRYGA, FOS, SPRY2, CRYGB, BDNF, SLC1A3, OR6C1, OR4C11, OR6P1, MKKS, SLC22A3, IL1B, CDH23, OR7A10, OR4F17, OR4M1, OR1D5, CBLN1, OR5V1, UBC, ABAT, OR4N2, PRKCZ, PCDHB14, COL2A1, RIMS1, OR4F4, PDE6B, MUSK, OR10G8, TAS2R46, AGT, DMD, AGRN, OR51V1, COL18A1, OR5P3, GABRA2, OR5M10, DLGAP2, OR5K2, OR10H1, SOD1, OR10H5, OR10H3, GABARAP, GJB2, CACNA2D4, OR4F6, EYS, OR3A2, OR51A2, OR51A4, WNT7A |
|  | GO:0050890 | cognition | 47 | 0.00 | PRKCZ, OR1A2, LXN, MYO7A, OR11A1, COL2A1, OR11H12, RIMS1, OR4F4, KIFC3, CRYGA, SPRY2, FOS, CRYGB, PDE6B, BDNF, OR10G8, SLC1A3, TAS2R46, OR6C1, OR4C11, OR6P1, MKKS, IL1B, OR7A10, CDH23, OR51V1, COL18A1, OR5P3, OR5M10, OR5K2, OR4F17, OR10H1, OR4M1, OR10H5, SOD1, OR1D5, OR10H3, GJB2, CACNA2D4, EYS, OR4F6, OR5V1, OR3A2, OR51A2, OR4N2, OR51A4 |
|  | GO:0010941 | regulation of cell death | 39 | 0.01 | DLC1, PRKCZ, HTATIP2, C6, IL19, RAG1, RRAGA, SOX4, COL2A1, PF4, ASNS, FOXO3, VDR, BDNF, AGT, IL1B, TOP2A, COL18A1, PGAP2, CARD8, ADAMTS20, MADD, MSH2, TGFBR1, RXRA, KLF10, BCL2A1, STRADB, SOD1, ADRB2, CUL4A, UBC, NLRP12, SERPINB2, ADAM17, ID3, NGFR, BMP7, LRRK2 |
|  | GO:0010604 | positive regulation of macromolecule metabolic process | 37 | 0.05 | DLC1, GLIS3, THRA, FOXA2, SOX4, PF4, FOXO3, RORA, CTNNB1, FOS, AGT, PSMB2, HINFP, IL1B, AGRN, NR2F2, KDM5A, SUPT5H, TGFBR1, TP53BP1, RXRA, FOXA1, MED14, UBE2N, ADRB2, PSMC6, ATF4, PKNOX1, PSMA5, IRF6, MED17, ETS2, UBC, FOXE1, ADAM17, BMP7, LRRK2 |
|  | GO:0007155 | cell adhesion | 33 | 0.02 | DLC1, PCDHA8, THRA, PCDHB14, CTNND1, ARF6, COL2A1, CRNN, PCDHGA4, CLDN12, CTNNB1, CDH6, LAMB3, AGT, PCDHA10, ANGPTL3, MLLT4, DSCAM, CDH23, COL18A1, SELP, PCDHGA10, CLCA2, PCDHGA11, PCDH9, AJAP1, NID2, MUC4, SIGLEC6, ITGB1BP1, CDON, TROAP, ADAM17 |
|  | GO:0022610 | biological adhesion | 33 | 0.02 | DLC1, PCDHA8, THRA, PCDHB14, CTNND1, ARF6, COL2A1, CRNN, PCDHGA4, CLDN12, CTNNB1, CDH6, LAMB3, AGT, PCDHA10, ANGPTL3, MLLT4, DSCAM, CDH23, COL18A1, SELP, PCDHGA10, CLCA2, PCDHGA11, PCDH9, AJAP1, NID2, MUC4, SIGLEC6, ITGB1BP1, CDON, TROAP, ADAM17 |
|  | GO:0007267 | cell-cell signaling | 32 | 0.00 | CXCL5, IL18, LTBP4, PCDHB14, CCL8, RIMS1, EPHB1, CTNNB1, PGR, SPRY2, MUSK, BDNF, SLC1A3, IFNA7, AGT, DMD, SLC22A3, IL1B, AGRN, MLLT4, GABRA2, DLGAP2, NUDT3, KLF10, FZD1, GABARAP, GJB2, CBLN1, SIGLEC6, UBC, ABAT, WNT7A |
|  | GO:0032989 | cellular component morphogenesis | 24 | 0.00 | COL18A1, RXRA, MYO7A, LIFR, RPL24, STRADB, SOD1, EPHB1, CTNNB1, SLITRK1, CRYGB, SLITRK4, BDNF, SLC1A3, DMD, DLX5, UBC, GBX2, MKKS, NGFR, BMP7, DCLK1, DSCAM, CDH23 |
|  | GO:0030030 | cell projection organization | 21 | 0.01 | RXRA, MYO7A, LIFR, ARF6, RPL24, SOD1, PRKG1, EPHB1, SLITRK1, BDNF, SLITRK4, DLX5, DMD, UBC, GBX2, MKKS, NGFR, BMP7, DCLK1, DSCAM, CDH23 |
|  | GO:0009628 | response to abiotic stimulus | 20 | 0.02 | COL18A1, TRPC3, LXN, MSH2, IL18, RBM3, TGFBR1, CRNN, SOD1, PDE6B, FOS, ADRB2, SLC1A3, GTF2H2C, AGT, DDB2, MKKS, IL1B, NGFR, IGFBP2 |
|  | GO:0044092 | negative regulation of molecular function | 20 | 0.01 | CAST, PRKCZ, THRA, NF2, ADCY5, GABBR1, RAG1, PDCD4, SPRY2, PSMC6, ADRB2, PSMA5, HEXIM1, PSMB2, GADD45G, UBC, IL1B, ID3, ANGPTL3, GADD45B |
|  | GO:0051726 | regulation of cell cycle | 19 | 0.02 | MSH2, MADD, SIPA1, DBC1, RPL24, ASNS, ATR, PDCD4, HEXIM1, GADD45G, HINFP, IL1B, ADAM17, CDK5RAP3, ID3, NGFR, GADD45B, BMP7, RAD17 |
|  | GO:0007423 | sensory organ development | 18 | 0.00 | MYO7A, RPL24, COL2A1, SOD1, EPHB1, CTNNB1, HESX1, CRYGA, CRYGB, SPRY2, BDNF, PKNOX1, DLX5, GBX2, MAB21L1, BMP7, DSCAM, CDH23 |
|  | GO:0001501 | skeletal system development | 17 | 0.04 | THRA, LGALS3, TGFBR1, KLF10, GABBR1, COL2A1, CTNNB1, VDR, COL9A2, HOXB7, KAZALD1, DLX5, ETS2, HOXC5, BMP7, WNT7A, TLL1 |
|  | GO:0043086 | negative regulation of catalytic activity | 17 | 0.01 | CAST, PRKCZ, NF2, ADCY5, GABBR1, RAG1, PDCD4, SPRY2, PSMC6, PSMA5, HEXIM1, PSMB2, GADD45G, UBC, IL1B, ANGPTL3, GADD45B |
|  | GO:0051094 | positive regulation of developmental process | 17 | 0.01 | SH3PXD2B, FOXA2, ADAMTS20, KLF10, FOXA1, PF4, FOXO3, CTNNB1, BDNF, ADRB2, AGT, DLX5, IL1B, NGFR, ANGPTL3, BMP7, WNT7A |
|  | GO:0060429 | epithelium development | 17 | 0.00 | COL18A1, DLC1, FOXA2, FOXA1, FZD1, CTNNB1, PGR, LCE1E, IRF6, AGT, DLX5, SPRR1B, LCE1D, MKKS, ID3, NR2F2, BMP7 |
|  | GO:0001944 | vasculature development | 16 | 0.01 | COL18A1, SELP, HTATIP2, IL18, TGFBR1, NCL, CTNNB1, CRKL, PKNOX1, DLL4, AGT, ECSCR, GBX2, IL1B, ANGPTL3, NR2F2 |
|  | GO:0031399 | regulation of protein modification process | 16 | 0.05 | DLC1, PRKCZ, NF2, TGFBR1, PDCD4, UBE2N, PSMC6, PSMA5, AGT, PSMB2, UBC, NLRP12, IL1B, ADAM17, BMP7, LRRK2 |
|  | GO:0048858 | cell projection morphogenesis | 16 | 0.01 | RXRA, LIFR, RPL24, EPHB1, SLITRK1, BDNF, SLITRK4, DLX5, DMD, UBC, GBX2, MKKS, NGFR, BMP7, DCLK1, DSCAM |
|  | GO:0003006 | developmental process involved in reproduction | 15 | 0.04 | MSH2, SOX3, FOXA1, FOXO3, SOD1, GJB2, CDH6, PGR, VDR, AGT, PIWIL1, NKX3-1, MKKS, HSD17B4, WNT7A |
|  | GO:0051240 | positive regulation of multicellular organismal process | 15 | 0.02 | PRKCZ, CARD8, IL18, PF4, SOD1, ADRB2, SLC1A3, AGT, UBC, NLRP12, TBXA2R, IL1B, ADAM17, BMP7, AGPAT2 |
|  | GO:0007398 | ectoderm development | 13 | 0.02 | FOXA2, NF2, CTNNB1, C1ORF68, LAMB3, LCE1E, IRF6, SPRR1B, LCE1D, FOXE1, NGFR, WNT7A, NSDHL |
|  | GO:0048568 | embryonic organ development | 13 | 0.01 | MYO7A, TGFBR1, SPINT1, COL2A1, SOD1, HESX1, SPRY2, HOXB7, DLX5, FOXE1, GBX2, BMP7, CDH23 |
|  | GO:0043062 | extracellular structure organization | 12 | 0.01 | COL18A1, MUSK, LGALS3, ADAMTS20, KAZALD1, AGT, TGFBR1, PCDHB14, COL2A1, AGRN, WNT7A, CTNNB1 |
|  | GO:0007548 | sex differentiation | 10 | 0.05 | PGR, MSH2, AGT, MKKS, HSD17B4, FOXO3, SOD1, WNT7A, CDH6, GJB2 |
|  | GO:0009266 | response to temperature stimulus | 10 | 0.00 | FOS, ADRB2, LXN, RBM3, IL18, AGT, IL1B, NGFR, CRNN, SOD1 |
|  | GO:0035239 | tube morphogenesis | 10 | 0.02 | DLC1, SPRY2, FOXA2, AGT, FOXA1, GBX2, NKX3-1, MKKS, BMP7, CTNNB1 |
|  | GO:0001763 | morphogenesis of a branching structure | 9 | 0.00 | SPRY2, FOXA2, AGT, FOXA1, GBX2, NKX3-1, SPINT1, BMP7, CTNNB1 |
|  | GO:0001819 | positive regulation of cytokine production | 9 | 0.01 | CARD8, IL18, AGT, NLRP12, ADAM17, IL1B, PF4, SOD1, AGPAT2 |
|  | GO:0043583 | ear development | 9 | 0.01 | HESX1, SPRY2, BDNF, DLX5, MYO7A, GBX2, COL2A1, SOD1, CDH23 |
|  | GO:0006414 | translational elongation | 8 | 0.04 | RPS25, SNORA7A, RPS29, RPL9, UBC, RPL24, RPL7A, RPL10A |
|  | GO:0007605 | sensory perception of sound | 8 | 0.03 | SPRY2, SLC1A3, MYO7A, MKKS, COL2A1, SOD1, CDH23, GJB2 |
|  | GO:0050954 | sensory perception of mechanical stimulus | 8 | 0.04 | SPRY2, SLC1A3, MYO7A, MKKS, COL2A1, SOD1, CDH23, GJB2 |
|  | GO:0009409 | response to cold | 5 | 0.00 | FOS, ADRB2, RBM3, IL18, AGT |
|  | GO:0021675 | nerve development | 5 | 0.01 | BDNF, SLC1A3, RPL24, NGFR, EPHB1 |
| **Decreased** | GO:0050890 | cognition | 39 | 0.01 | OR51D1, OBP2A, OR8H3, CACNB2, TAS1R3, TIMM13, COMT, OR10G2, RIMS1, OR4F3, OR2V2, CALCA, PRKAR2B, CRYGC, BEST1, OR7G3, MCOLN3, OR8A1, IMPDH1, OR51V1, S100P, OR2AG2, OR5M10, MYO3A, FSCN2, HTT, OR8G2, OR4M2, OR52E4, PDE6G, VSX1, RGS9BP, SFRP5, TAS2R16, CRH, OR6V1, OR4N2, WDR1, CACNA1C |
|  | GO:0007600 | sensory perception | 33 | 0.04 | OR51D1, OBP2A, OR8H3, CACNB2, TAS1R3, TIMM13, OR10G2, RIMS1, OR4F3, OR2V2, CALCA, CRYGC, OR7G3, BEST1, MCOLN3, OR8A1, IMPDH1, OR51V1, OR2AG2, OR5M10, MYO3A, FSCN2, OR8G2, OR4M2, OR52E4, PDE6G, VSX1, RGS9BP, SFRP5, TAS2R16, OR6V1, OR4N2, WDR1 |
|  | GO:0010629 | negative regulation of gene expression | 25 | 0.01 | BCLAF1, CBX2, PDX1, TNFRSF4, CALCA, MEIS2, WWP2, SND1, DDX20, NRG1, NR2F2, ZNF423, DMBX1, ZNF593, EHMT1, VHL, ARID5B, TP53, FOSB, SIRT7, MBD2, FOXP1, BRCA1, SIRT3, IRF7 |
|  | GO:0006259 | DNA metabolic process | 23 | 0.03 | EXO1, RBBP4, UVRAG, TP53, PAPD5, BRCA1, FOXP1, GTF2H2, GLRX2, ATRX, POLD3, CCDC111, DNASE1, FANCM, CRY2, CSNK1E, KRT7, PSIP1, TOP3A, PMS2CL, FANCA, DNAJA3, REPIN1 |
|  | GO:0010817 | regulation of hormone levels | 11 | 0.01 | UGT1A8, LY6E, HTT, GHRH, CHST8, CYP26B1, CRH, COMT, PDX1, CACNA1C, BCO2 |
|  | GO:0010639 | negative regulation of organelle organization | 7 | 0.03 | TRIOBP, ARHGAP6, TRIM54, BUB1, ADD2, BRCA1, HDAC6 |
|  | GO:0009914 | hormone transport | 5 | 0.05 | LY6E, HTT, GHRH, PDX1, CACNA1C |

**Supplementary Table 6. Summary of mRNA-Seq alignment. AD, Alzheimer’s disease patient; C, control normal subject.**

| **Case** | **Total**  **reads** | **Filtered reads** | **Used**  **reads** | **Mapped reads** | **Uniquely mapped**  **reads** | **Mapping rate** | **Depth (fold)** |
| --- | --- | --- | --- | --- | --- | --- | --- |
| AD1 | 73,765,170 | 4,486,971 | 69,278,199 | 57,479,883 | 54,675,736 | 0.83 | 70.40 |
| AD2 | 46,649,484 | 7,437,142 | 39,212,342 | 27,720,699 | 26,556,784 | 0.71 | 34.83 |
| AD3 | 89,221,024 | 5,066,386 | 84,154,638 | 73,507,453 | 69,998,152 | 0.87 | 91.09 |
| AD4 | 83,992,030 | 4,417,273 | 79,574,757 | 69,804,655 | 66,284,130 | 0.88 | 86.72 |
| AD5 | 51,885,624 | 4,050,321 | 47,835,303 | 41,198,357 | 39,363,034 | 0.86 | 51.06 |
| AD6 | 42,808,104 | 2,862,916 | 39,945,188 | 35,093,955 | 34,130,508 | 0.88 | 43.39 |
| C1 | 86,560,836 | 4,096,283 | 82,464,553 | 74,746,342 | 72,362,323 | 0.91 | 96.21 |
| C2 | 63,522,360 | 2,232,081 | 61,290,279 | 55,521,159 | 53,528,720 | 0.91 | 71.85 |
| C3 | 64,903,408 | 4,465,589 | 60,437,819 | 53,282,648 | 50,998,086 | 0.88 | 68.42 |
| C4 | 106,904,762 | 5,107,121 | 101,797,641 | 91,294,910 | 88,502,485 | 0.9 | 116.01 |
| C5 | 88,466,194 | 4,063,825 | 84,402,369 | 76,614,124 | 74,160,506 | 0.91 | 98.78 |
| C6 | 67,737,612 | 4,202,735 | 63,534,877 | 56,972,366 | 55,446,855 | 0.9 | 73.06 |

**Supplementary Table 7. Differentially expressed genes between AD patients and normal subjects identified by EdgeR and DESeq (Please confer an attached excel file including Supplementary Table 4 and 7).**

**Supplementary Table 8. GOBPs represented by the DEGs. 'Up-regulated' and 'Down-regulated' represent the GOBPs represented by the up- and down-regulated genes in AD, respectively. 'Count' means the number of the genes involved in the corresponding GOBP. Such genes are listed. FE, fold enrichment.**

| **Change** | **Term**  **ID** | **Description** | **Count** | **FE** | **P-**  **value** | **Genes** |
| --- | --- | --- | --- | --- | --- | --- |
| **Up-regulated** | GO:0022610 | biological adhesion | 105 | 1.72 | 0.00 | ITGB3BP, DLC1, LYPD3, MMRN1, AZGP1, CD44, TGFBI, CDH26, CDH23, CLCA2, ROCK1, EFNB1, CDHR4, CDHR5, CERCAM, BCL2L11, CTNNA3, NCAM1, JUP, F5, TESK2, TGFB1I1, MFAP4, DST, TNFRSF12A, NINJ2, DSCAML1, ITGB5, NINJ1, ITGB3, ITGB1, PXN, ITGBL1, ALCAM, LAMB2, ITGAX, ITGB8, SPP1, COL18A1, BMP1, LPP, ITGA2, MCAM, ECM2, LAMA2, COL14A1, DSG2, ITGA5, PKP4, ABL1, AEBP1, PCDHA9, CLDN9, MYBPC1, FER, SRPX, WISP1, DLG5, CYR61, CNTNAP4, TRPM7, INPPL1, CPXM2, DDR1, SLC26A6, PSEN1, CNTN2, VCAN, SEMA4D, AOC3, MIA, TNC, COL3A1, CDH1, NEO1, CLDN11, CLDN15, VCAM1, CD9, ANXA9, COL6A6, FAT4, COL27A1, COL6A3, MYBPH, COL6A2, ACAN, PSTPIP1, PKD2, CD22, GP1BA, COL8A1, AMICA1, THBS2, COL8A2, HAPLN2, MAG, NFASC, HSPG2, COL4A6, CLDN23, ERBB2IP, CDH19, DSC3, MUC16 |
|  | GO:0007155 | cell adhesion | 105 | 1.73 | 0.00 | ITGB3BP, DLC1, LYPD3, MMRN1, AZGP1, CD44, TGFBI, CDH26, CDH23, CLCA2, ROCK1, EFNB1, CDHR4, CDHR5, CERCAM, BCL2L11, CTNNA3, NCAM1, JUP, F5, TESK2, TGFB1I1, MFAP4, DST, TNFRSF12A, NINJ2, DSCAML1, ITGB5, NINJ1, ITGB3, ITGB1, PXN, ITGBL1, ALCAM, LAMB2, ITGAX, ITGB8, SPP1, COL18A1, BMP1, LPP, ITGA2, MCAM, ECM2, LAMA2, COL14A1, DSG2, ITGA5, PKP4, ABL1, AEBP1, PCDHA9, CLDN9, MYBPC1, FER, SRPX, WISP1, DLG5, CYR61, CNTNAP4, TRPM7, INPPL1, CPXM2, DDR1, SLC26A6, PSEN1, CNTN2, VCAN, SEMA4D, AOC3, MIA, TNC, COL3A1, CDH1, NEO1, CLDN11, CLDN15, VCAM1, CD9, ANXA9, COL6A6, FAT4, COL27A1, COL6A3, MYBPH, COL6A2, ACAN, PSTPIP1, PKD2, CD22, GP1BA, COL8A1, AMICA1, THBS2, COL8A2, HAPLN2, MAG, NFASC, HSPG2, COL4A6, CLDN23, ERBB2IP, CDH19, DSC3, MUC16 |
|  | GO:0042981 | regulation of apoptotic process | 100 | 1.43 | 0.00 | DLC1, ITGB3BP, STAT5A, TGFB3, TNFSF15, TNFSF14, GDNF, TGFB1, PTGIS, CD44, CDKN2C, PRAME, ROCK1, ACTN4, CRYAB, RELA, PIM3, BCL2L11, NME3, ALOX15B, TIAL1, TXNDC5, MYO18A, TMX1, GSDMA, ADORA2A, ERBB3, NFKBIA, ADA, SLC11A2, PRUNE2, BMF, TRAF4, RUNX3, PLAGL2, FGD4, HIP1, COL18A1, CFLAR, MUC2, GNRH1, SPHK1, NR4A1, GAS1, ATM, P2RX7, NOTCH1, UACA, SFRP1, HDAC1, IKBKB, ABL1, NUAK2, INS-IGF2, PREX1, MITF, PAWR, BOK, AEN, POU4F1, DLG5, ALX4, IP6K2, SGK3, ARHGEF7, SMO, TNFRSF9, HIPK1, PSEN1, HIPK2, SORT1, FOXC2, NAIP, NGFR, SEMA4D, CLN8, ALOX12, MAP3K11, C6, CDH1, SFN, ZBTB16, POU3F3, BCL6, INPP5D, PCSK6, PHLDA3, TXNIP, ZFP91-CNTF, MSH6, BIRC7, IGF2, ADIPOQ, PLEKHF1, CNTF, DUSP1, NUPR1, ID3, TIAF1, IGFBP3, TP53INP1, DNM2 |
|  | GO:0007010 | cytoskeleton organization | 67 | 1.77 | 0.00 | DLC1, TLN1, NUAK2, CROCC, PREX1, WASF2, RHOQ, CNP, TTN, RHOU, OFD1, CTTNBP2, NDE1, DES, ANG, GSN, FMNL2, ACTN4, ROCK1, TRPM7, FOXJ1, CRYAB, ERMN, PROX1, VASP, FLNA, PLCE1, TESK2, CLN8, DST, CXCL1, SHROOM1, CAV1, HAUS5, SHROOM4, TCAP, CALD1, SIPA1, ELN, ANLN, MYO9B, RCC1, ITGB1, DAAM2, CDC42EP2, MACF1, TEKT1, KRT8, CNN2, BCL6, TEKT4, WIPF1, CNN1, TRIP10, FGD4, INF2, SUN2, RICTOR, CAPN3, SEMA6A, ERBB2IP, MYH11, MAP7, SYNM, KPTN, ABL1, ARAP1 |
|  | GO:0007423 | sensory organ development | 31 | 1.56 | 0.01 | TCAP, ERBB3, MITF, TGFB3, PRRX1, ZIC1, TGFB1, DFNB31, ECE1, CHD7, COL8A1, COL8A2, CDH23, MAF, CRYAB, SIX2, SIX5, DLL1, TBX1, NR4A3, GAS1, PROX1, BCL2L11, ASCL1, DDR1, SP1, SIX1, TGIF1, FOXC2, TGIF2, CLN8 |
|  | GO:0051960 | regulation of nervous system development | 31 | 1.86 | 0.00 | TF, ZNF488, EFNA1, TNFRSF12A, REST, TGFB1, MBP, METRN, C11ORF9, NKX6-2, S1PR5, POU3F2, CDK5RAP2, NKX2-2, SPP1, ZFP91-CNTF, MAG, ARHGEF1, PLXNB1, RELA, DLL1, ISL1, ASCL1, SMO, NOTCH1, CNTF, PSEN1, SIX1, TGIF1, TGIF2, SEMA4D, NGFR |
|  | GO:0009100 | glycoprotein metabolic process | 27 | 1.54 | 0.03 | GYPC, HS3ST5, SPOCK3, GALNT6, FUT7, KEL, FUT6, TSPAN8, POMT2, B3GNT8, TRAK2, ST3GAL4, B3GNT6, ACAN, CHST14, FUT2, PCSK6, GAL3ST1, KLK6, LIPA, CHST3, ST6GALNAC2, MGAT1, MAN2A1, EGFLAM, PSEN1, LRP2 |
|  | GO:0016055 | Wnt receptor signaling pathway | 21 | 1.82 | 0.01 | DIXDC1, LZTS2, NKD1, RYK, MITF, TLE4, TAX1BP3, FZD5, FRZB, WISP1, MACF1, SFRP1, SFRP2, FBXW4, SFRP4, LRP6, HBP1, TGFB1I1, WNT6, AXIN1, LRP5 |
|  | GO:0007229 | integrin-mediated signaling pathway | 16 | 2.63 | 0.00 | PLP1, COL3A1, ITGA2, ITGB5, ITGB3, ITGB1, ITGBL1, ADAMTS7, DOCK1, GAB2, ITGAX, ERBB2IP, ITGB8, ITGA5, ADAM33, DST |
|  | GO:0007265 | Ras protein signal transduction | 16 | 1.75 | 0.04 | PLD1, ARHGEF1, ROCK1, WASF2, RTKN, MYO9B, ABCA1, MAPKAPK2, TAX1BP3, RHOU, PLCE1, CDC42EP1, RASGRP3, COL1A2, RAPGEF6, RHOG |
|  | GO:0006643 | membrane lipid metabolic process | 13 | 1.85 | 0.05 | ACER3, SGPL1, A4GALT, SPTLC2, SGMS2, FA2H, SPHK1, PSAPL1, ASAH2, P2RX7, ITGB8, CLN8, GAL3ST1 |
|  | GO:0000302 | response to reactive oxygen species | 13 | 2.00 | 0.03 | TXNIP, GNAO1, CRYAB, RELA, DUOX1, PXN, ADA, FOS, EP300, DUSP1, SERPINE1, COL1A1, FANCC |
| **Down-regulated** | GO:0050877 | neurological system process | 125 | 1.61 | 0.00 | SYT1, GRIK1, GABRB3, SYT5, GRIK2, STRC, SNCA, CNGB1, VIPR1, CALB1, BDNF, IL1B, SCN2B, BAIAP3, GRIN2A, MYLK2, TIMM8B, PITPNM3, CAMK4, AKAP5, UNC13C, RBP4, DRD2, DRD5, CACNB2, RIMS1, GAD2, B3GNT1, GAD1, PLAT, GABRD, GABRA2, CPNE6, GABRA6, GABRA5, LIN7B, AFG3L2, VDAC3, NPY5R, VDAC1, PDE7B, NPY, PENK, PRKAR1B, ADRA1B, CHRNB2, CACNA1E, FABP7, CORT, SST, CRYM, ADRA1D, CACNA1B, LXN, TRPV2, TACR1, UCHL1, GABBR2, PAX2, KCNIP2, KCNIP1, CRYBB1, SLC1A4, SLC1A2, KCNQ3, SLC1A6, DLG4, SHC3, SLC1A1, EGR1, KCNMA1, STX1A, EGR3, EGR2, CNTN5, CCKBR, NRXN3, ALDH5A1, PI4KA, PRKCG, CDK5, STX1B, GNAL, GRM8, PPEF1, CHRM1, OTOF, PSEN2, RAB3A, PPFIA3, CPLX1, NDN, TH, GNG13, TIMM10, OXTR, TAC1, KIT, FKBP1B, DTNBP1, HRH1, PDE1B, SYN1, NPTX2, SYN2, PPP3CB, HTR3A, SNAP25, NEFL, APBA1, GUCA1A, DLGAP1, DLGAP2, ATP1A3, GJB6, PARK2, GRIA4, PRPH2, SLC17A7, SLC17A8, PNOC, GRIA1, CRH, HTR2C, HTR2A |
|  | GO:0019226 | transmission of nerve impulse | 84 | 3.73 | 0.00 | SYT1, GRIK1, GABRB3, SYT5, GRIK2, SNCA, GABBR2, KCNIP2, VIPR1, KCNIP1, SLC1A4, SLC1A2, KCNQ3, SLC1A6, DLG4, SHC3, SLC1A1, KCNMA1, STX1A, EGR3, EGR2, SCN2B, ALDH5A1, BAIAP3, NRXN3, GRIN2A, PI4KA, MYLK2, STX1B, CDK5, CAMK4, GRM8, CHRM1, AKAP5, UNC13C, RAB3A, PPFIA3, CPLX1, DRD2, DRD5, TH, OXTR, TAC1, CACNB2, FKBP1B, RIMS1, HRH1, GAD2, SYN1, NPTX2, SYN2, PPP3CB, HTR3A, GAD1, SNAP25, APBA1, GABRD, PLAT, DLGAP1, GABRA2, DLGAP2, CPNE6, GABRA6, GABRA5, LIN7B, PARK2, GRIA4, AFG3L2, VDAC3, NPY5R, VDAC1, SLC17A7, PDE7B, PNOC, NPY, GRIA1, CRH, CACNA1E, CHRNB2, CORT, HTR2C, SST, HTR2A, CACNA1B |
|  | GO:0055114 | oxidation-reduction process | 59 | 1.44 | 0.00 | LDHA, KCNAB2, SNCA, NDUFAB1, PRDX2, HSD11B1L, COX5A, UQCRFS1, GLDC, NDUFS5, MSRA, NDUFS4, ALOX12B, SRD5A1, ASPH, SARDH, PCYOX1L, ALDH5A1, MICAL2, F8, NDUFC2, CYP4X1, DHRS2, UQCRH, FDX1L, STEAP2, MECR, MDH2, MDH1, NDUFB3, ME1, ME3, NDUFB6, TH, KMO, CYP26B1, IDH2, HSD17B6, DMGDH, SDR16C5, BDH1, TYW1B, CYP2B7P1, HSD17B8, GLRX, NDUFA4, NDUFA5, NOX5, NDUFA8, CYP46A1, CYP2C8, NDUFA7, DHRS11, HGD, BLVRA, CYP4A11, VAT1L, DIO2, MPO |
|  | GO:0030182 | neuron differentiation | 57 | 2.02 | 0.00 | GPRIN1, TUBB2B, TUBB2A, UCHL1, KCNIP2, PAX2, LUZP6, CDC42, WNT1, BDNF, ATXN10, PAX7, ROBO2, SPON2, TUBB3, KCNMA1, EGR2, EFNB3, STMN2, NRXN3, PTPRR, CDK5, SLIT1, NUMBL, NDEL1, RND1, GAP43, KALRN, RAB3A, CDK5R1, CCK, LPPR4, NDN, RTN4RL1, DRD2, TH, NNAT, HPRT1, LINGO1, TUBB, PTK2B, MTPN, B3GNT1, LHX6, RTN4RL2, LAMB1, NEFL, SNAP25, DCLK1, IL6, GNAO1, BHLHE22, NTNG1, AFG3L2, DLX1, DLX5, MAP2, CHRNB2 |
|  | GO:0051726 | regulation of cell cycle | 33 | 1.55 | 0.01 | CDK5R1, CDK5R2, NEK2, EDN1, MOV10L1, PIN1, ADCYAP1, CDC42, DIRAS3, OVOL2, NPM2, CDKN2D, BUB1, CAMK2D, IL1B, RANBP1, ZWILCH, CDC7, CDK1, FAM5B, CENPF, MYLK2, INHA, CDKN3, CDK5, PKIA, PLK4, MAD2L1, CCND2, ZWINT, BRE, MYO16, GADD45A |
|  | GO:0007611 | learning or memory | 31 | 4.34 | 0.00 | DRD2, DRD5, TACR1, TH, OXTR, TAC1, KIT, CALB1, BDNF, PDE1B, DLG4, IL1B, SHC3, SNAP25, EGR1, EGR2, GABRA5, ATP1A3, GRIN2A, PRKCG, PARK2, CDK5, VDAC3, VDAC1, GRIA1, PRKAR1B, PSEN2, ADRA1B, CRH, CHRNB2, HTR2A |
|  | GO:0042493 | response to drug | 26 | 1.87 | 0.00 | YWHAZ, STAR, DRD2, SNCA, HMGCS1, OXTR, TIMP4, UQCRFS1, PCSK1, SLC1A2, BDNF, PTK2B, IL1B, SRD5A1, RAB6A, GNAO1, ATP1A3, GRIN2A, CENPF, STAT1, NME1, FABP3, SST, HTR2C, HTR2A, SLC46A2 |
|  | GO:0006836 | neurotransmitter transport | 22 | 4.12 | 0.00 | SYT1, PPFIA3, RAB3A, CPLX2, ICA1, GABRA2, STX1A, CPLX1, BAIAP3, NRXN3, LIN7B, PARK2, SLC6A17, STX1B, RIMS1, SLC17A7, SLC32A1, SLC17A8, SYN1, SYN2, SV2B, SNAP25 |
|  | GO:0009416 | response to light stimulus | 17 | 1.92 | 0.02 | GUCA1A, RBP4, DRD2, DRD5, GRIN2A, ATP1A3, CNGB1, KIT, CDK5, GTF2H2, SLC1A2, RPAIN, PDE1B, CDKN2D, ADRA1B, CHRNB2, TUBB3 |
|  | GO:0048511 | rhythmic process | 15 | 1.82 | 0.04 | KCNMA1, EGR3, EGR2, DRD2, GRIN2A, OXTR, TIMP4, INHA, VGF, NPY5R, CCND2, CRH, CHRNB2, ATOH7, FSHB |
|  | GO:0019933 | cAMP-mediated signaling | 15 | 2.46 | 0.00 | MCHR1, PTGER3, DRD2, DRD5, FPR1, P2RY12, PTHLH, CRHR1, GNAL, NDUFS4, ADRA1B, MC4R, RAPGEF4, CORT, ADRA1D |
|  | GO:0048015 | phosphatidylinositol-mediated signaling | 14 | 2.47 | 0.00 | PTGER3, CCKBR, DRD2, DRD5, TACR1, EDN1, PI4KA, GNG13, HRH1, NPY, CHRM1, ZWINT, HTR2C, HTR2A |
|  | GO:0007200 | phospholipase C-activating G-protein coupled receptor signaling pathway | 11 | 3.29 | 0.00 | HRH1, PTGER3, CCKBR, DRD2, TACR1, DRD5, CHRM1, EDN1, GNG13, HTR2C, HTR2A |
|  | GO:0007215 | glutamate receptor signaling pathway | 8 | 4.98 | 0.00 | CDK5R1, GRIK1, SSTR1, GRIK2, ATP1A3, GRIN2A, GRIA3, GRIA4 |
|  | GO:0042417 | dopamine metabolic process | 7 | 5.44 | 0.00 | SNCB, DRD2, TH, SNCA, GRIN2A, PARK2, HPRT1 |
|  | GO:0007212 | dopamine receptor signaling pathway | 6 | 5.18 | 0.00 | GNAL, CALY, GNAO1, DRD2, DRD5, HMP19 |

**Supplementary Table 9. Quantitative PCR (q-PCR) primer sequences for determining the relative expression of H3K9me3-landscaped epigenome.**

| **Gene** | **Sequence** |
| --- | --- |
| *BDNF* | F: GCTGCAAACATGTCCATGAG |
|  | R: GTAACCCATGGGATTGCACT |
| *GABBR1* | F: AAGAGTGTGTCCACTGAGAA |
|  | R: AGGAGGAGAAAACTATGGCA |
| *GABRA2* | F: TCGAATTCAGGATGATGGGA |
|  | R: AGGAGCAACCTGTACTGAAT |
| *GPRASP1* | F: TCTGTTAAGACACCCTGGTT |
|  | R: ACTCCAAATGGTCTTCATGC |
| *HIST2H2BE* | F: AAAAAGGGCTCCAAGAAAGC |
|  | R: TCGAAGATGTCGTTGACGAA |
| *ID3* | F: ACGACATGAACCACTGCTAC |
|  | R: CGTTGGAGATGACAAGTTCC |
| *KIF1C* | F: ACTCGGAGAAGGTCAGTAAG |
|  | R: AGAGTCCCTGTAGGGGATAA |
| *NCALD* | F: CAGAGCATGAGATCCAGGAA |
|  | R: TTCCCCCTCGAAGTTACACT |
| *SEPT4* | F: GACAAGGAGTATGTGGGCTT |
|  | R: CCACTGCATGCTTAGTGATC |
| *SYT12* | F: TTGGCATCGATGAGGATGAG |
|  | R: TGAGGTTCTTGGCCTTAACC |

**
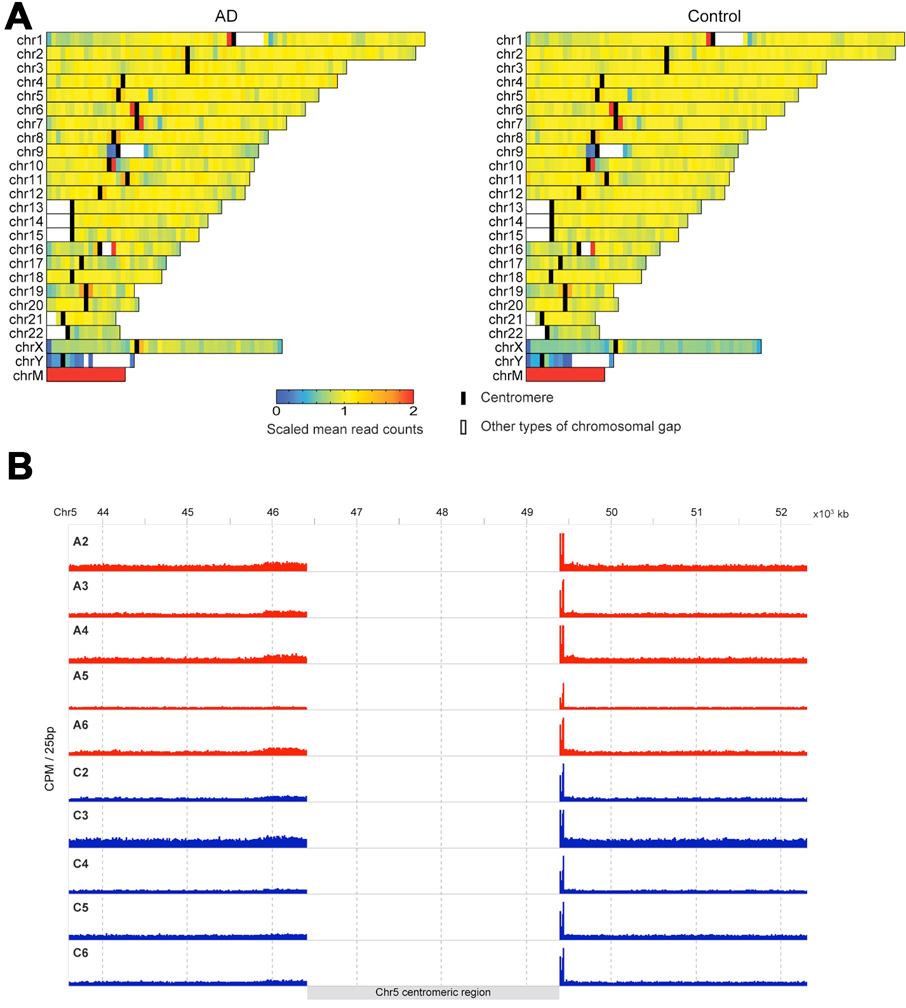
**

**Supplementary Figure 1. The occupancy of H3K9me3 is altered in AD.** (**A**) The change of H3K9me3 enrichment on chromosomes of brain samples from AD patients (AD) and normal control subjects (Control). (**B**) The occupancy of H3K9me3 on the centromere of chromosome 5. IGV plots were derived from each AD patient (A2-A5) and normal control subject (C2-C6).

**
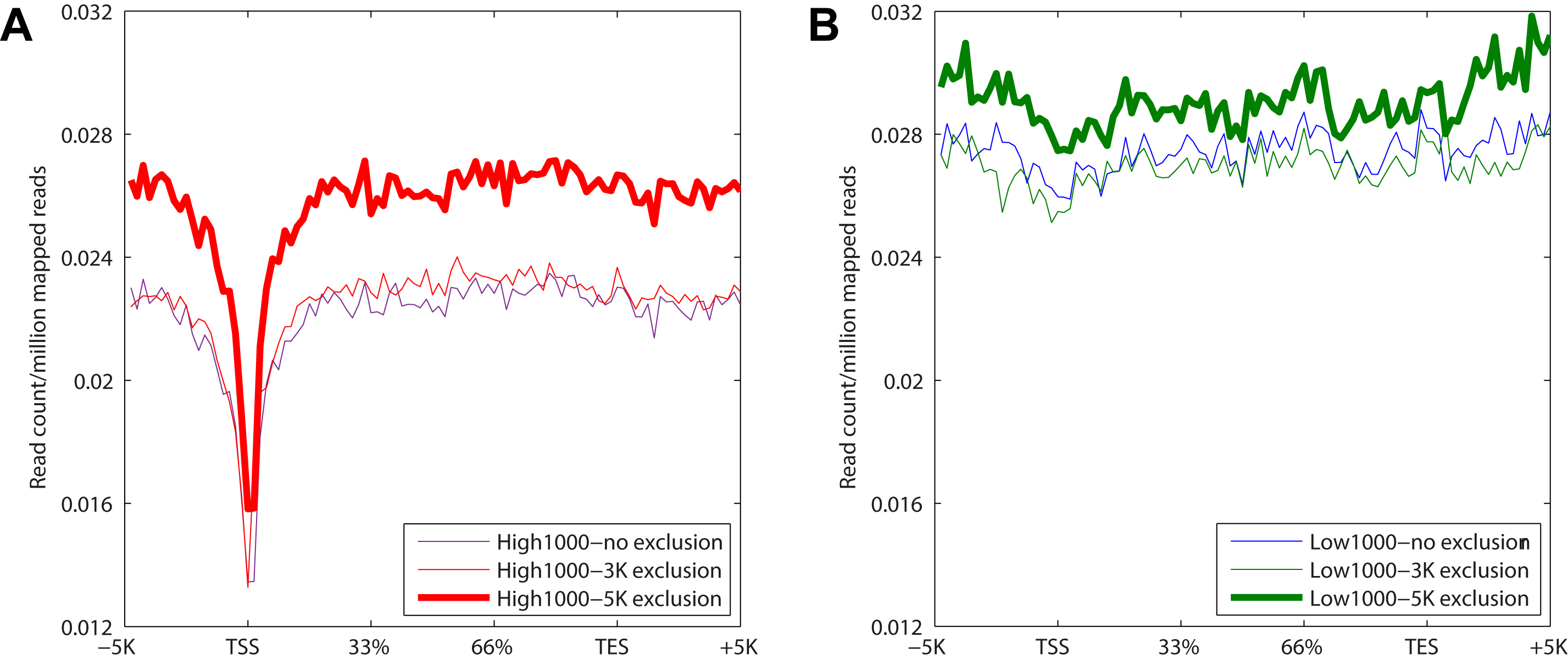
**

**Supplementary Figure 2. Expression stratified association analysis.** H3K9me3 profiles of the two sets of genes with high (**A**) and low (**B**) mRNA expression levels, respectively. In the analysis for each set of genes, three kinds of gene exclusion were used: 1) no exclusion was done (no exclusion) and 2-3) genes shorter than 3K (3K exclusion) and 5K (5K exclusion) were excluded.

**
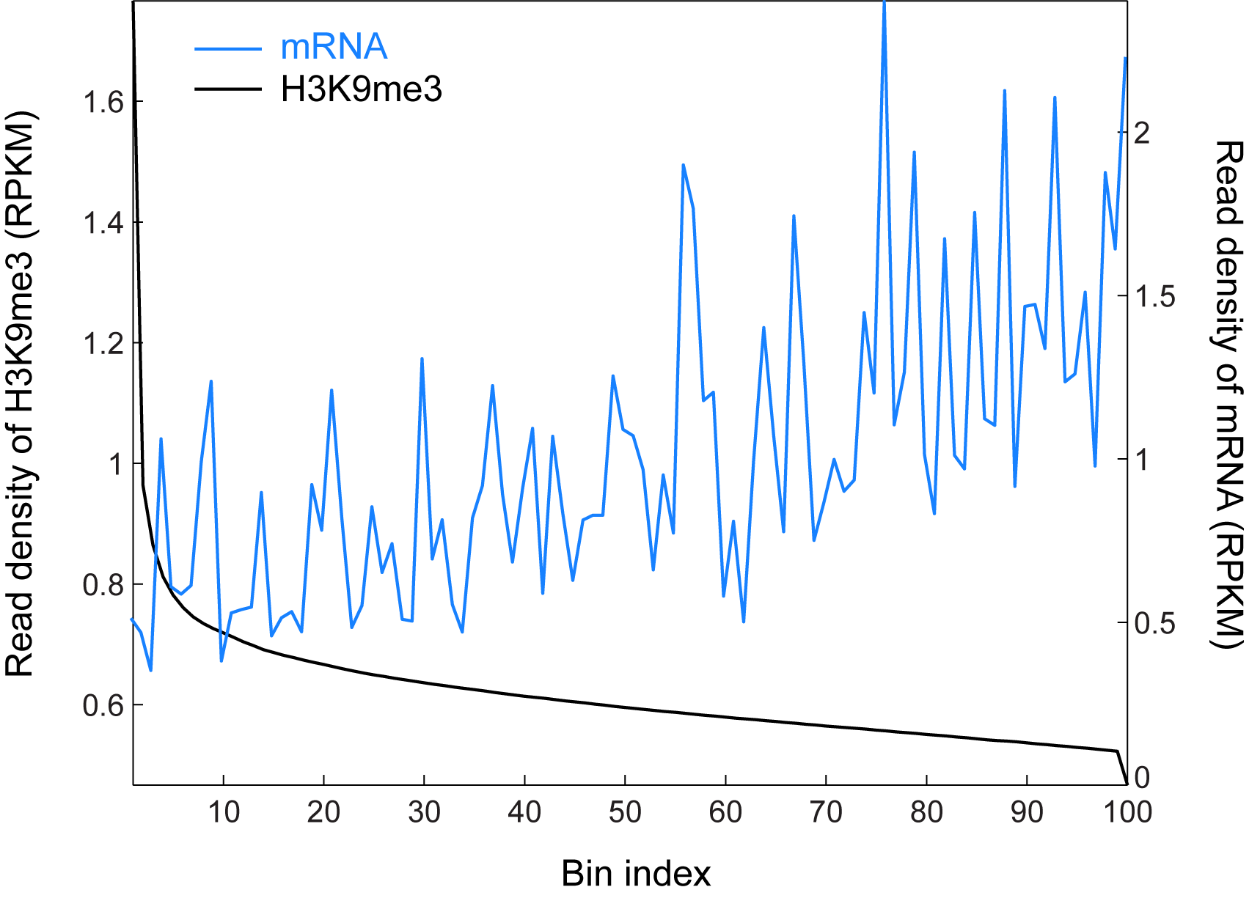
**

**Supplementary Figure 3. An inverse correlation between the enrichment of H3K9me3 (black line) and the expression level of mRNA (blue line) is found in the cortex of AD patients.**

**­
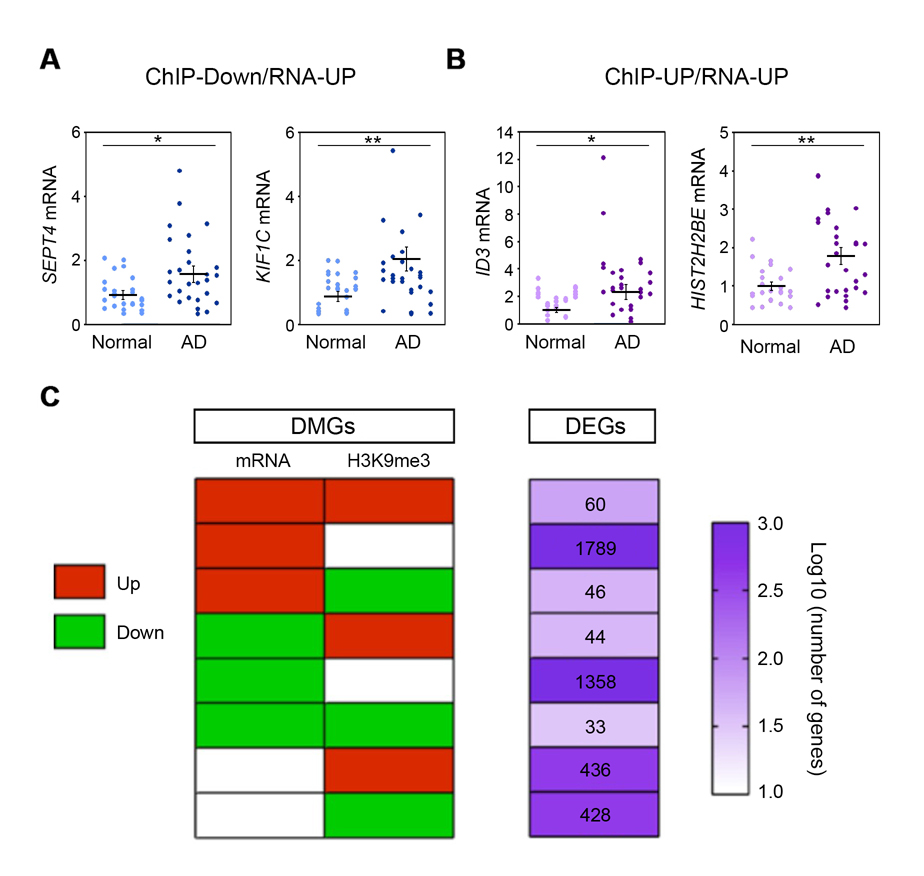
**

**Supplementary Figure 4.** **Quantitative real-time PCR (qPCR) verifies that H3K9me3-landscaped transcriptomes are altered in AD.** (**A**) *SEPT4* and *KIF1c* were up regulated in AD patients (N=20) compared to normal subjects (N=20). The occupancy of H3K9me3 in the promoter regions of these genes was lower in AD. Significantly different at **, *p* <0.001. (**B**) *ID3* and *HIST2H2BE* were up regulated in AD patients (n=20) compared to normal subjects (n=20). The occupancy of H3K9me3 in the promoter regions of these genes was higher in AD. Significantly different at **, *p*<0.001. (**C**) Eight groups of DMGs and DEGs were analyzed and presented in the heat map.


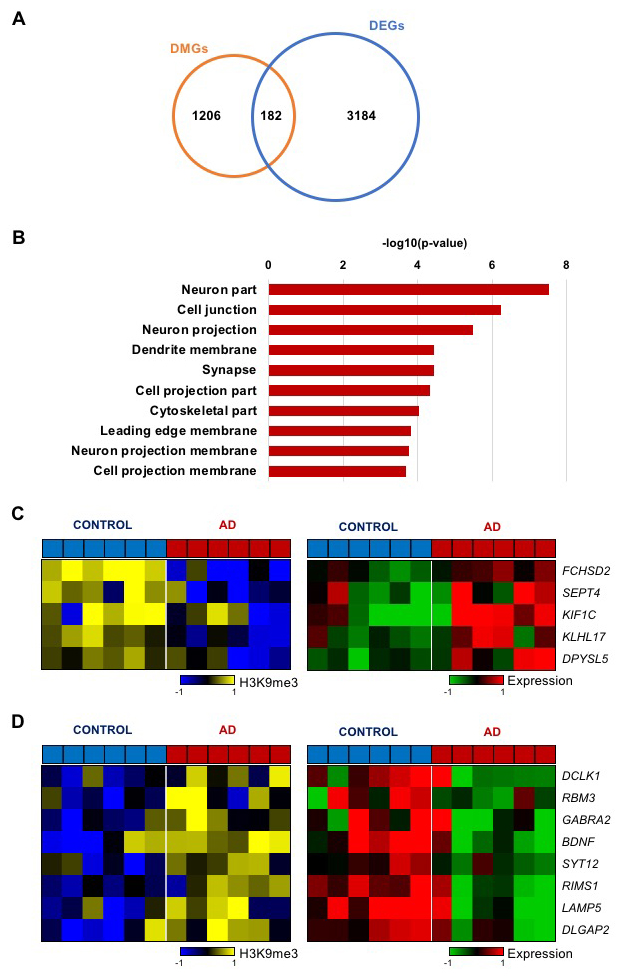


**Supplementary Figure 5**. **H3K9me3-landscaped epigenome signatures are differentially modulated in a cell-type-specific manner**. (A) Venn diagram summarizing the association between differentially H3K9me3-occupied genes (DMGs) and differentially expressed genes (DEGs). (B) Top 10 GO enrichment analysis of common genes between DMGs and DEGs. Neuron part pathway was shown to be highly enriched in AD. (C) Heatmap depicting the hypomethylated (low level of H3K9me3) and up-regulated genes in neuron part pathway. (D) Heatmap representing the hypermethylated (high level of H3K9me3) and down-regulated genes in neuron part pathway


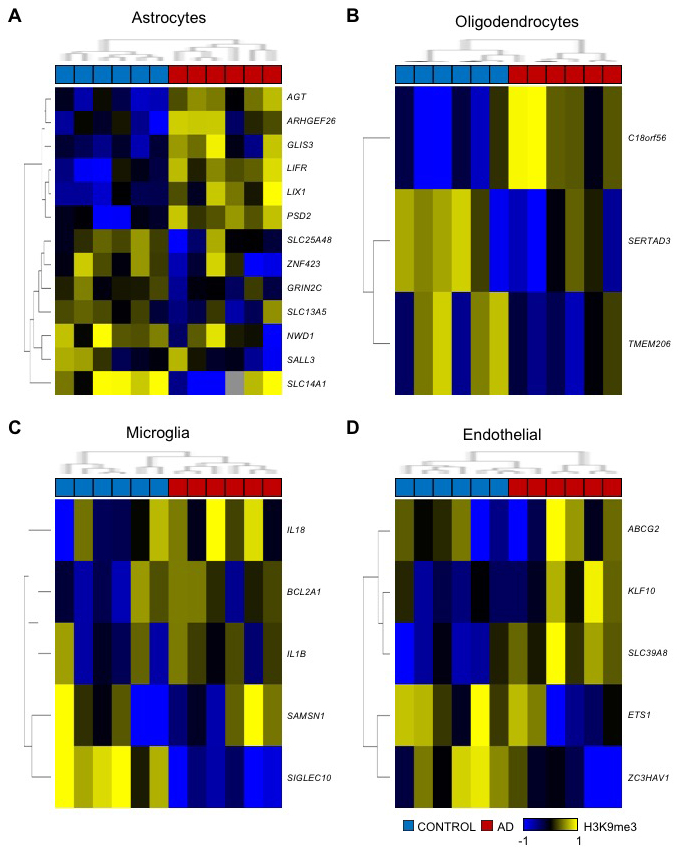


**Supplementary Figure 6.** **Cell-type-specific genes sorted by H3K9me3 occupancy (DMG)**. (A) Astrocytes, (B) Oligodendrocytes, (C) Microglia, and (D) Endothelial cells. The cell-type-specific genes were marked in the Supplementary Table 4 and 7.


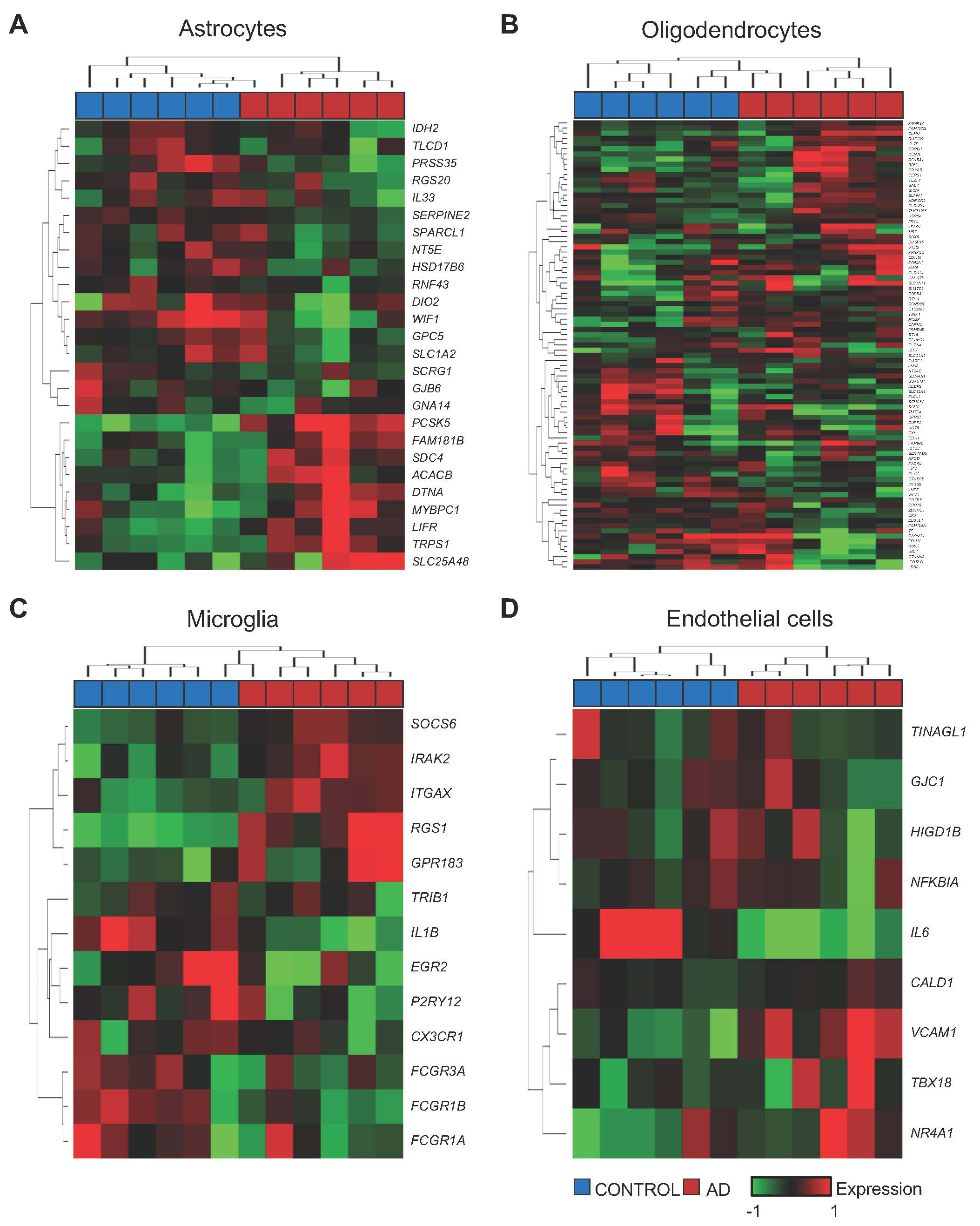


**Supplementary Figure 7.** **Cell-type-specific genes sorted by mRNA expression level (DEG)**. (A) Astrocytes, (B) Oligodendrocytes, (C) Microglia, and (D) Endothelial cells. The cell-type-specific genes were marked in the Supplementary Table 4 and 7.


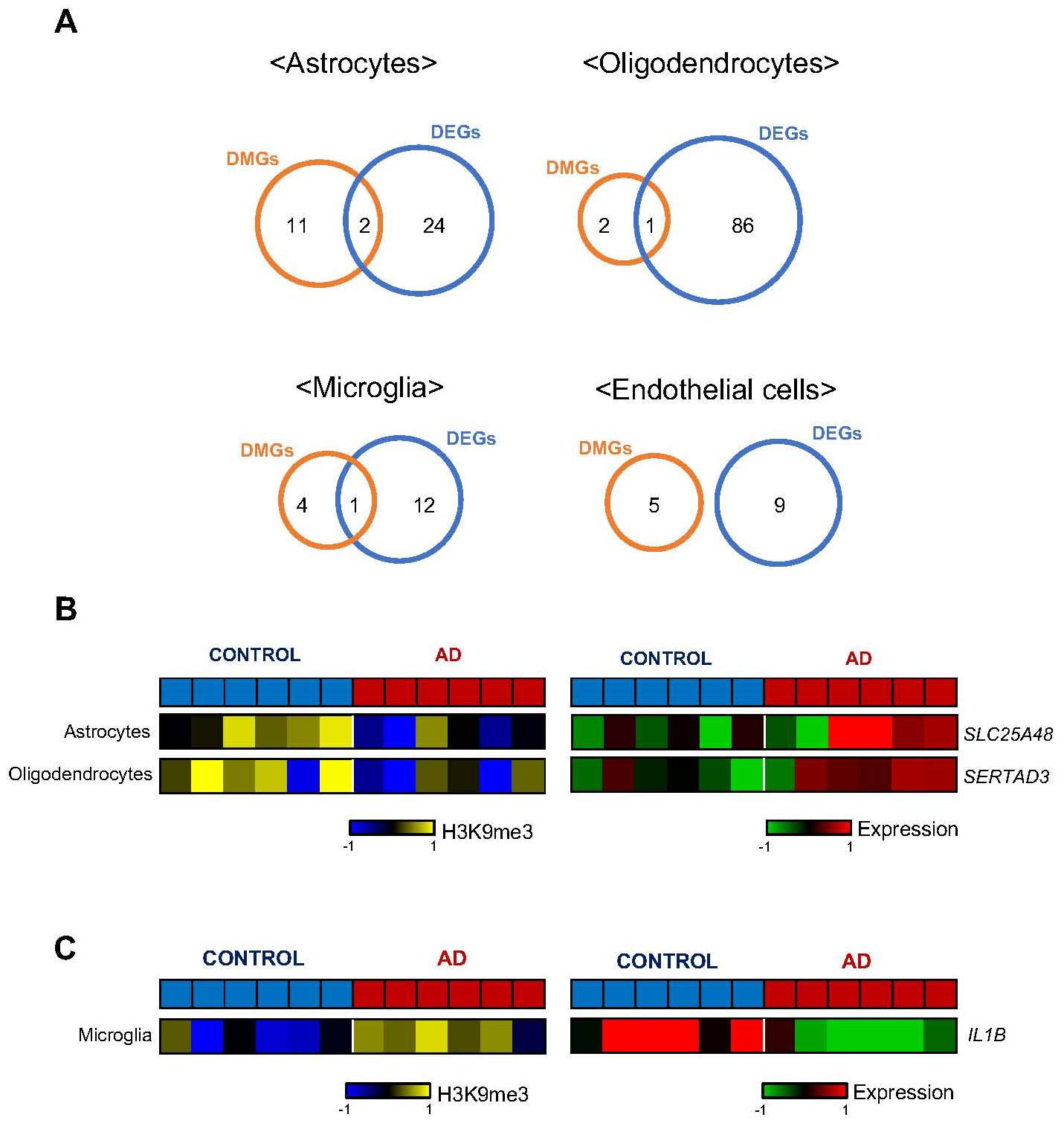


**Supplementary Figure 8.** **H3K9me3-landscaped epigenome signatures are differentially modulated in a cell-type (astrocyte, oligodendrocyte, and microglia)-specific manner.** (A) Comparison of the number of differentially methylated genes (DMGs) and differentially expressed genes (DEGs) (B) Heatmaps depicting the hypomethylated (left) and up-regulated genes (right) of cell-type-specific genes (astrocytes and oligodendrocytes). (C) Heatmap representing the hypermethylated (left) and down-regulated genes (right) in microglia.

**
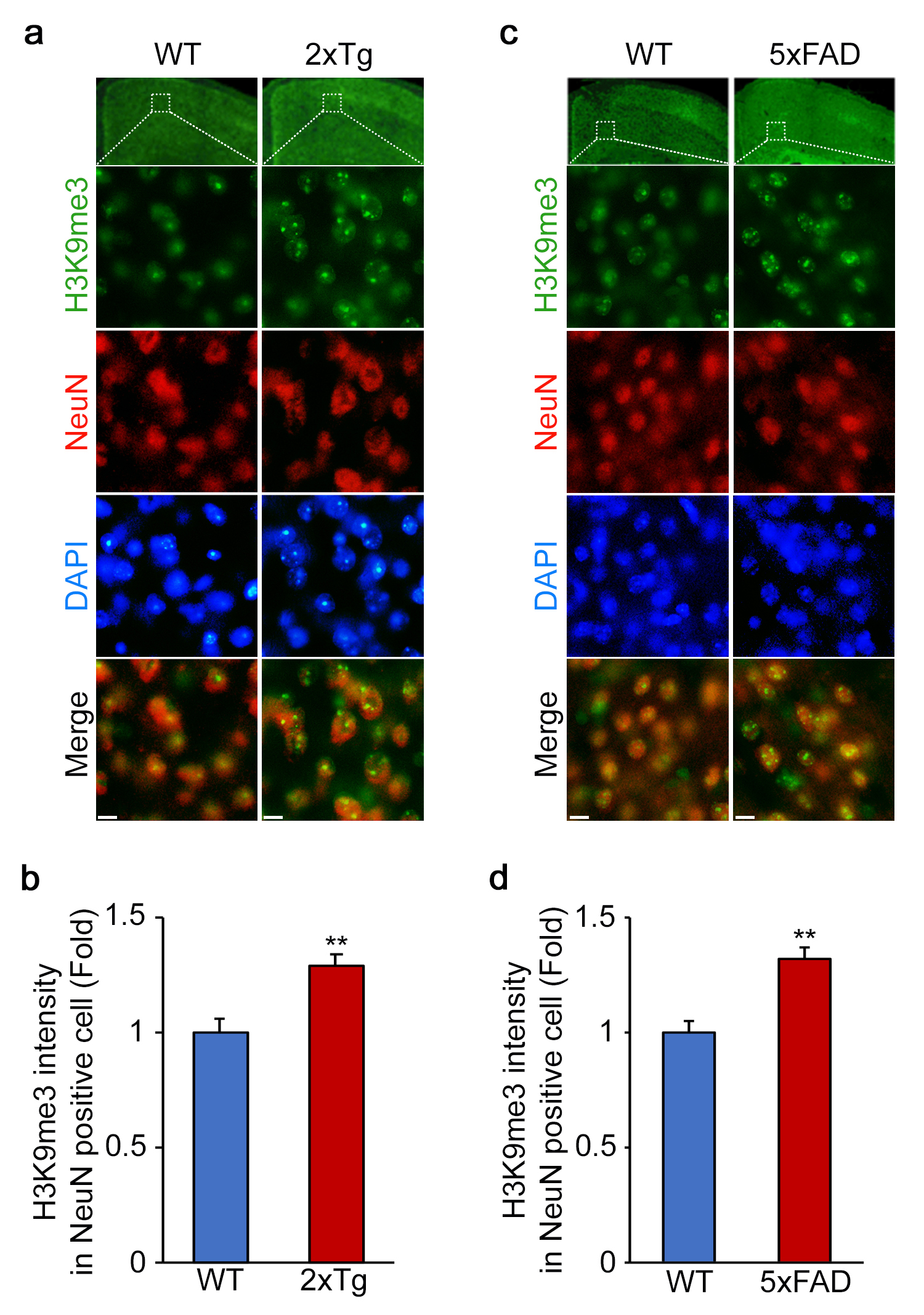
**

**Supplementary Figure 9. H3K9me3-positive heterochromatin structure is altered in AD transgenic mouse models.** (A and B) Confocal microscopy image and densitometry analysis showed that the intensity of H3K9me3-positive heterochromatin was significantly increased in the cortex of APP/PS1 mutant mice (n=3) compared to WT mice (n=3). (C and D) H3K9me3-postive chromatin condensation was increased in the cortex of 5xFAD mice (n=3) compared to WT mice (n=3). **, Significantly different from WT at *p* < 0.01.

**
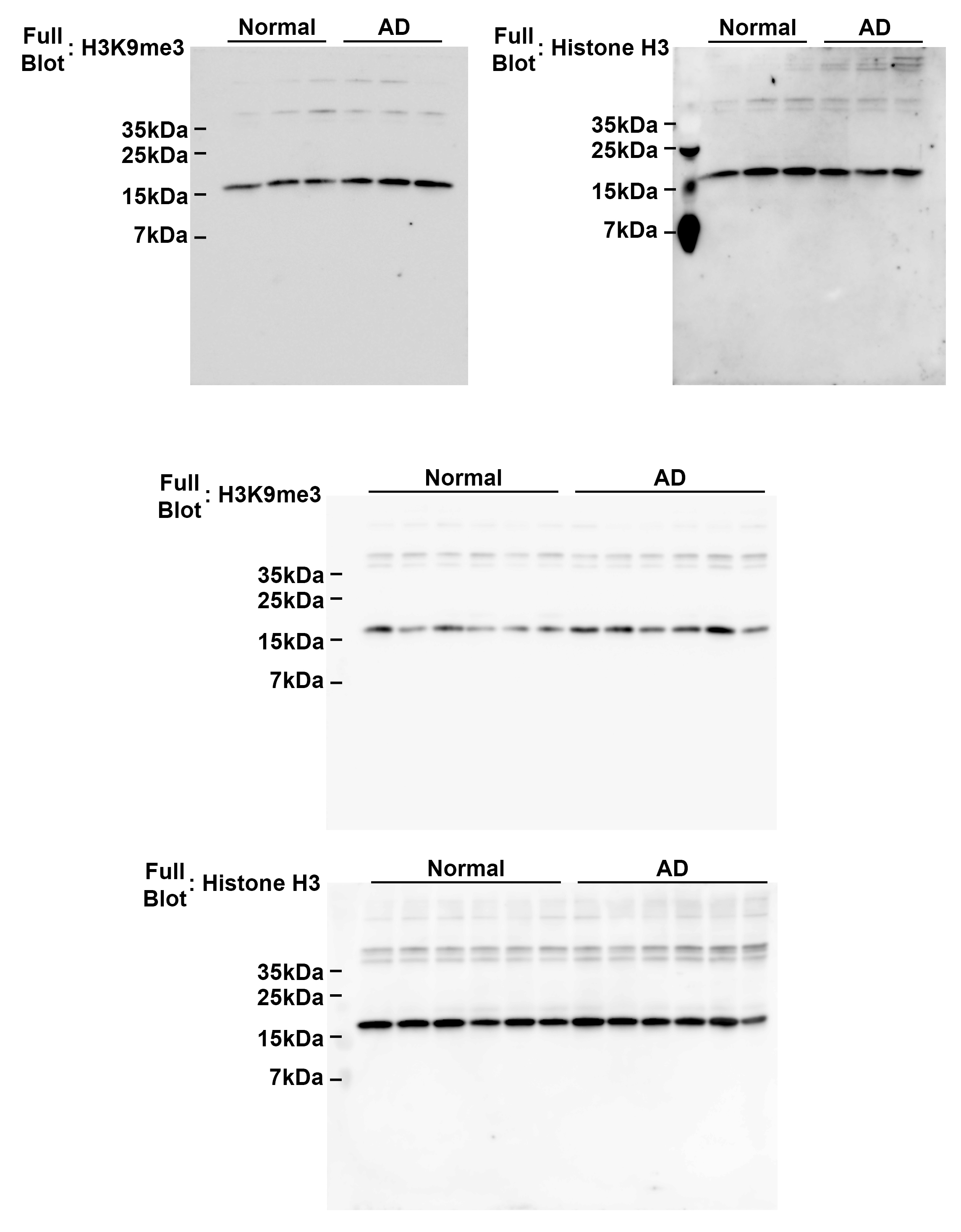
**

**Supplementary Figure 10. The whole Western blot image of H3K9me3 and histone H3 those are presented in the main Figure 1.**
